# Resuscitation With Placental Circulation Intact Compared With Cord Milking: A Randomized Clinical Trial

**DOI:** 10.1001/jamanetworkopen.2024.50476

**Published:** 2024-12-13

**Authors:** Simone Pratesi, Martina Ciarcià, Luca Boni, Stefano Ghirardello, Cristiana Germini, Stefania Troiani, Eleonora Tulli, Miria Natile, Gina Ancora, Giovanni Barone, Stefania Vedovato, Federica Bertuola, Francesca Parata, Giovanna Mescoli, Fabrizio Sandri, Roberta Corbetta, Luisa Ventura, Giulia Dognini, Flavia Petrillo, Luigia Valenzano, Raffaele Manzari, Anna Lavizzari, Fabio Mosca, Iuri Corsini, Chiara Poggi, Carlo Dani

**Affiliations:** 1 Careggi University Hospital, Department of Neuroscience, Psychology, Drug Research and Child Health, University of Florence, Firenze, Italy; 2 Clinical Trials Coordinating Center, Clinical Epidemiology Unit, IRCCS Ospedale Policlinico San Martino, Genova, Italy; 3 SC Terapia Intensiva Neonatale e Neonatologia Fondazione IRCCS Policlinico San Matteo, Pavia, Italy; 4 Department of Pediatrics, Santa Maria della Misericordia Hospital, Perugia, Italy; 5 Neonatal Intensive Care Unit, Division of Neonatology, Infermi Hospital, Rimini, Italy; 6 Department of Pediatrics, Neonatal Intensive Care Unit, San Bortolo Hospital, Vicenza, Italy; 7 Neonatal Intensive Care Unit, Department of Women’s and Children’s Health, Maggiore Hospital, Bologna, Italy; 8 Neonatal Intensive Care Unit, Fondazione IRCCS San Gerardo dei Tintori, Monza, Italy; 9 Neonatal Intensive Care Unit, Department of Women’s and Children’s Health, Di Venere Hospital, Bari, Italy; 10 Department of Mother and Infant Science, Fondazione IRCCS Ca’ Granda Ospedale Maggiore Policlinico, University of Milan, Milano, Italy; 11 Division of Neonatology, Careggi University Hospital, Firenze, Italy

## Abstract

**Importance:**

Among preterm newborns undergoing resuscitation, delayed cord clamping for 60 seconds is associated with reduced mortality compared with early clamping. However, the effects of longer durations of cord clamping with respiratory support are unknown.

**Objective:**

To determine whether resuscitating preterm newborns while keeping the placental circulation intact and clamping the cord after a long delay would improve outcomes compared with umbilical cord milking.

**Design, Setting, and Participants:**

This randomized clinical trial (PCI Trial) was conducted at 8 Italian neonatal intensive care units from April 2016 through February 2023 and enrolled preterm newborns born between 23 weeks 0 days and 29 weeks 6 days of gestation from singleton pregnancies.

**Interventions:**

Enrolled newborns were randomly allocated to receive at-birth resuscitation with intact placental circulation for 180 seconds or umbilical cord milking followed by an early cord clamping (within 20 seconds of life).

**Main Outcomes and Measures:**

The primary outcome was the composite end point of death, grade 3 to 4 intraventricular hemorrhage, and bronchopulmonary dysplasia at 36 weeks of postconception age. Prespecified secondary end points were the single components of the composite primary outcome. An intention-to-treat analysis was conducted.

**Results:**

Of 212 mother-newborn dyads who were randomized, 209 (median [IQR] gestational age, 27 [26-28] weeks; median [IQR] birth weight, 900 [700-1070] g) were enrolled in the intention-to-treat population; 105 were randomized to the placental circulation intact group, and 104 were randomized to the cord milking group. The composite outcome of death, grade 3 to 4 intraventricular hemorrhage, or bronchopulmonary dysplasia occurred in 35 of 105 newborns (33%) in the placental circulation intact group vs 39 of 104 newborns (38%) in the cord milking group (odds ratio, 0.83; 95% CI, 0.47-1.47; *P* = .53).

**Conclusions and Relevance:**

In a randomized clinical trial of preterm newborns at 23 to 29 weeks’ gestational age, intact placental resuscitation for 3 minutes did not lower the composite outcome of death, grade 3 to 4 intraventricular hemorrhage, or bronchopulmonary dysplasia compared with umbilical cord milking.

**Trial Registration:**

Clinicaltrials.gov Identifier: NCT02671305

## Key Points

**Question:**

Can resuscitation of preterm newborns with the placental circulation intact improve their clinical outcomes compared with umbilical cord milking?

**Findings:**

In this randomized clinical trial that included 209 newborns, the composite risk of death, grade 3 to 4 intraventricular hemorrhage, and bronchopulmonary dysplasia at 36 weeks of postconception age was not statistically significantly different between newborns resuscitated with placental circulation intact and those resuscitated with umbilical cord milking.

**Meaning:**

These findings suggest that, compared with umbilical cord milking, resuscitation with placental circulation intact for 3 minutes does not improve outcomes.

## Teaser

This randomized clinical trial examines whether resuscitating preterm newborns while keeping the placental circulation intact and clamping the umbilical cord after a long delay would improve outcomes vs umbilical cord milking.

## Introduction

Delayed cord clamping (of 30-60 seconds) improves outcomes in preterm newborns not requiring immediate resuscitation at birth compared with immediate cord clamping.^1,2,3^ The American Heart Association and American Academy of Pediatrics and European international guidelines for neonatal resuscitation suggest that for preterm newborns who do not require resuscitation at birth, it is reasonable to delay cord clamping for longer than 30 seconds.^4,5,6^ The majority of preterm newborns worldwide continue to receive an immediate cord clamping after birth, in particular, if delivered by cesarean delivery, to ensure a rapid start of resuscitation.^7,8,9,10^

Physiological studies^11,12^ on animals indicate that effectively ventilating the lungs before clamping the cord results in a smoother hemodynamic postnatal adaptation compared with immediately clamping the cord. Effective ventilation of the lungs after a very premature birth is probably achieved after a longer time than 30 to 60 seconds, both in spontaneously breathing preterm newborns and in those who require respiratory support.^13,14^ We hypothesized that a longer delay in clamping the cord while starting to assist the newborn should allow smoother cardiorespiratory adaptation, especially in newborns who are not immediately breathing at birth.

Umbilical cord milking (UCM) is considered an alternative procedure to be performed at birth when delayed cord clamping is not feasible (eg, if immediate resuscitation is needed), because it better benefits preterm newborns compared with immediate cord clamping.^3,6,15,16,17,18^ Unfortunately, to our knowledge, there are no studies comparing the effect of resuscitation with placental circulation intact (PCI) and delayed cord clamping with resuscitation with UCM in preterm newborns. Thus, the objective of our study was to compare the clinical outcomes observed with these 2 different strategies of resuscitation in preterm newborns.

## Methods

### Study Design

This trial followed the Consolidated Standards of Reporting Trials (CONSORT) reporting guidelines. The study protocol has been carried out following the principles of the Declaration of Helsinki^19^ and has been approved by the local ethical committees of all participating centers. All sites had approval for prenatal informed consent forms. Enrollment took place between April 2016 and February 2023. The trial protocol and statistical analysis plan are available in Supplement 1. This randomized, open-label, parallel-group, multicenter, prospective clinical trial, involving 8 neonatal intensive care units in Italy, evaluated the efficacy of PCI, compared with UCM, for improving outcomes in preterm newborns. Women at risk of preterm birth were offered participation and, if both parents accepted, they were given a written informed consent form. Immediately before an imminent delivery, a neonatologist allocated the newborn to 1 of the 2 treatment groups (PCI or UCM) in a 1:1 ratio, using a web-based randomization system^20^ and a minimization algorithm with gestational age class as stratification factor (23 weeks 0 days to 26 weeks 6 days vs 27 weeks 0 days to 29 weeks 6 days).

Compliance was defined as full adherence to the protocol. Compliance with the protocol was ensured by some procedures included in the site setup. The local principal investigator participated in preparatory meetings in which details of the study protocol and data collection were accurately discussed. All centers received detailed instructions on study procedures and web-based recording data. All centers did not have any experience of resuscitation with an intact cord prior to conducting this trial. To enhance compliance, all centers had to train on PCI before enrollment began, both by simulating on mannequins and performing it during delivery in older patients.

The study was not blinded, and the staff performing the study also cared for the newborns. Echocardiography and cerebral ultrasonography assessments were made by clinicians other than investigators involved in patient care, and researchers assessing study end points were blinded to the study treatments.

### Inclusion and Exclusion Criteria

Eligible newborns were those with a gestational age between 23 weeks 0 days and 29 weeks 6 days, whose parents signed the informed consent form. Exclusion criteria were twin or multiple births, placental and cord abnormalities, major congenital malformations, hydrops fetalis, and maternal severe compromise at delivery.

### Interventional Protocol

Patients who met the inclusion criteria at the time of birth were resuscitated following the current guidelines of the American Academy of Pediatrics.^4,5,6,21^ In the UCM group, 20 cm of the intact cord was squeezed over 2 seconds, repeated for a total of 4 times, and then the cord was clamped and cut within 20 seconds of life, whereas in the PCI group the cord was clamped at 180 seconds without milking. We chose to investigate a time-based cord clamping after 180 seconds of life because (1) we consider it a sufficient time to reach effective ventilation of preterm lungs, (2) placental transfusion seemed to reach a plateau after approximately 180 seconds,^22^ and (3) neonatologists are used to performing time-based steps during delivery room newborn resuscitation. Newborns of both groups received tactile stimulation before cord clamping. At birth, newborns were positioned supine on a portable resuscitation trolley (Lifestart; Inspiration Healthcare) in the PCI group and on the trolley or mother’s legs in the UCM group. Resuscitation of the newborn, if needed, took place on a standard newborn warmer in the UCM group, or portable resuscitation trolley in the PCI group. PCI and UCM were performed by holding the newborn at or within 10 cm below the level of the incision, both in vaginal and cesarean deliveries. If it was not possible to perform the assigned procedure, the newborn received an immediate umbilical cord clamping at birth. In both vaginal and cesarean deliveries, the neonatal resuscitation team consisted of a neonatologist and a nurse. A third person (midwife or second neonatologist or resident) was available in case a full resuscitation was needed. PCI during cesarean delivery has been described in more detail in a previous report.^23^ To maintain normothermia, at birth newborns were laid supine on a warming mattress, wrapped with a plastic bag up to the shoulders without drying, and the head was immediately covered with a cap. Neonatal care was begun with a fraction of inspired oxygen of 0.30, and oxygen was then titrated following the saturation of peripheral oxygen target for the minutes of life. Spontaneously breathing newborns with a suboptimal saturation of peripheral oxygen were assisted with nasal continuous positive airway pressure, whereas newborns not breathing at birth were stimulated and, if necessary, supported with positive pressure ventilation using a T-piece ventilator (Neopuff Infant T-Piece Resuscitator; Fisher & Paykel). Heart rate was monitored immediately after birth by pulse oximetry (Radical-7; Masimo Corporation) and by frequent auscultation of the newborn’s chest during the first minute of life.

### Outcomes

The primary outcome of the trial was the incidence of death or grade 3 to 4 intraventricular hemorrhage (IVH) or bronchopulmonary dysplasia (BPD) at 36 weeks of postconception age. Prespecified secondary end points were the single components of the composite primary outcome.

Other secondary data collected included the need for mechanical ventilation, the peak hemoglobin level and hematocrit in the first 24 hours of life, the need for and number of blood transfusions, necrotizing enterocolitis, and periventricular leukomalacia. The diagnosis of BPD was based on the definition of moderate and severe BPD by Jobe et al,^24^ IVH was diagnosed with the criteria of Papile et al,^25^ necrotizing enterocolitis was diagnosed with the criteria of Bell et al,^26^ and periventricular leukomalacia was diagnosed according to the criteria of de Vries et al.^27^

### Sample Size Calculation

When the present trial was designed, the incidence of the composite primary outcome in the 8 participating Italian neonatal intensive care units was approximately 47% (mortality, 15%; grade 3-4 IVH, 8%; and BPD, 24%). Meta-analyses showed a reduction of 58% in in-hospital mortality^28^ and of approximately 40% in any IVH^28,29^ in preterm newborns assisted at birth with delayed cord clamping or UCM instead of immediate cord clamping. Al-Wassia et al^30^ indicated that UCM could reduce BPD by 58% compared with immediate cord clamping or delayed cord clamping. No data on differences in outcomes between UCM and delayed cord clamping were available, although ventilating the newborn’s lungs during a prolonged delay in cord clamping, as with the PCI procedure, seemed to be the optimal approach at birth in preterm newborns.^12,31^ We considered as clinically relevant a relative reduction of the frequency of the composite primary outcome of at least 40%, equivalent to a 19% absolute difference between control and experimental groups. Considering a dropout rate of 5%, we calculated that 106 newborns should be enrolled in each group to detect this difference with 80% power at a significant level of *P* < .05 using a 2-sided χ^2^ test for heterogeneity.

### Statistical Analysis

The primary efficacy analysis was conducted on an intention-to-treat (ITT) basis, and the per-protocol (PP) approach for a secondary sensitivity analysis was used. Clinical characteristics of newborns in PCI and UCM groups were described using mean (SD), median (IQR), or frequencies and percentages. We verified the distribution of baseline patient characteristics across the participating centers by calculating the Cohen *d* standardized differences. The *d* index was never equal to or less than 0.10, demonstrating a good balance of such distributions.

Univariable statistical analysis was performed using the Wilcoxon rank-sum test for continuous variables and the χ^2^ test or Fisher exact test when appropriate for categorical variables. A 2-sided *P* < .05 was considered statistically significant. Relative effect estimates were expressed as odds ratio with Wald 95% CI. With a post hoc explorative intent, we evaluated the heterogeneity of the results within the 2 groups of newborns stratified by the type of delivery. Statistical analysis was performed with SAS statistical software version 9.4 (SAS Institute).

## Results

A total of 212 mother-newborn dyads were randomized between April 2016 and February 2023 (Figure). Three were excluded after randomization (2 stillbirths and 1 delivered outside the study window), leaving 209 enrolled patients in the intention-to-treat population (median [IQR] gestational age, 27 [26-28] weeks; median [IQR] birth weight, 900 [700-1070] g); 105 were randomized to the PCI group and 104 were randomized to the UCM group. The demographics of mothers and newborns were similar between the 2 groups and well balanced across the participating centers (Table 1). Five of 104 newborns in the UCM group and 20 of 105 newborns in the PCI group did not receive the assigned treatment, leaving 99 and 85 patients in the PP population. The mean (SD) time from birth to umbilical cord clamping in the PCI group was 139 (68) seconds in newborns included in the ITT analysis (105 newborns), and 163 (44) seconds in newborns included in the PP analysis (85 newborns). All newborns in the UCM group had their umbilical cord clamped before 20 seconds. Twenty percent of newborns in the PCI group (20 newborns) received early cord clamping. Clinicians were not able to follow the protocol for 30% of the PCI newborns (33 newborns).

**Figure.**
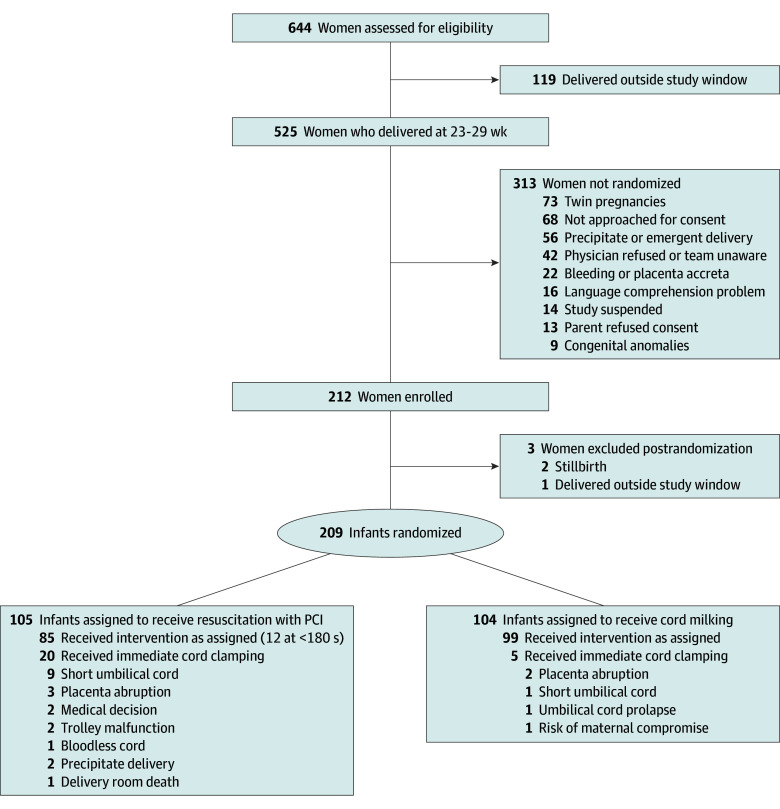
Patient Recruitment and Randomization Flowchart PCI indicates placental circulation intact.

**Table 1.** Maternal and Neonatal Characteristics by Treatment Group

Characteristic	Patients, No. (%)	
	UCM (n = 104)	PCI (n = 105)
Gestational age at birth, mean (SD), wk	26.6 (1.7)	26.7 (1.7)
23 wk 0 d to 26 wk 6 d	45 (43)	43 (41)
27 wk 0 d to 29 wk 6 d	59 (57)	62 (59)
Weight, mean (SD), g	898 (270)	942 (246)
Sex		
Female	45 (43)	45 (43)
Male	59 (57)	60 (57)
Cesarean delivery	64 (62)	54 (51)
Premature rupture of membranes	39 (38)	33 (31)
Chorioamnionitis	14 (13)	19 (18)
Preeclampsia	22 (21)	18 (17)
Intrauterine growth restriction	22 (21)	18 (17)
Steroids given before delivery	100 (96)	102 (97)
General anesthesia	10 (10)	11 (10)
Maternal age, mean (SD), y	32.1 (7.9)	32.4 (7.3)
Maternal sepsis	2 (1.9)	4 (3.8)
Maternal death	0	0
Maternal postpartum bleeding (>500 mL)	12 (11.5)	7 (6.6)
Apgar score at 1 min, median (IQR)	6 (5-7)	6 (5-7)
Apgar score at 5 min, median (IQR)	8 (7-9)	8 (7-9)
DR fraction of inspired oxygen maximum, median (IQR), %	50 (30-100)	50 (40-100)
DR positive pressure ventilation and/or nasal continuous positive airway pressure	96 (92)	96 (91)
DR intubation	26 (25)	26 (25)
Clinical Risk Index for Babies II score, median (IQR)	10 (8-12)	9 (8-12)
Body temperature at admission, median (IQR), °C	36.4 (36.0-36.7)	36.2 (35.8-36.6)

Abbreviations: DR, delivery room; PCI, placental circulation intact; UCM, umbilical cord milking.

Newborns assisted with UCM or PCI had a similar composite outcome (39 of 104 newborns [38%] in the UCM group vs 35 of 105 newborns [33%] in the PCI group; odds ratio, 0.83; 95% CI, 0.47-1.47; *P* = .53). The incidence of the composite outcome was higher in the subgroup of newborns born at 23 to 26 weeks of gestation compared with newborns born at 27 to 29 weeks of gestation, but did not vary between UCM and PCI groups (Table 2). The incidence of death (9 newborns [9%] in the PCI group and 13 newborns [13%] in the UCM group), grade 3 to 4 IVH (8 newborns [8%] in the PCI group and 12 newborns [12%] in the UCM group), and BPD (24 newborns [23%] in the PCI group and 25 newborns [24%] in the UCM group) were similar in the UCM and PCI groups (Table 3). We found that among newborns born at 23 to 26 weeks of gestation the occurrence of grade 3 to 4 IVH was similar in PCI compared with the UCM group (7 newborns [16%] vs 11 newborns [24%]). Among the secondary outcomes, there were no differences in the need for mechanical ventilation, the peak of hemoglobin and hematocrit in the first 24 hours, and the need for blood transfusions between the groups (Table 4). The PP analysis confirmed the results of the ITT analysis (eTables 1-3 in Supplement 2). In particular, the PP analysis of our data showed a reduction (from 16% [7 newborns] to 7% [2 newborns]) in the incidence of death in the subgroup of newborns born at 23 to 26 weeks of gestation in the PCI group but not in the UCM group. The PP analysis of our data showed a reduction (from 16% [7 newborns] to 10% [3 newborns]) of the incidence of 3 to 4 IVH in the subgroup of newborns born at 23 to 26 weeks of gestation in PCI and not in the UCM group.

**Table 2.** Primary Composite Outcome Overall and in Prespecified Subgroups for Intention to Treat Analysis

Outcome	Newborns, No./total No. (%)		OR (95% Cl)
	UCM (n = 104)	PCI (n = 105)	
Overall	39/104 (38)	35/105 (33)	0.83 (0.47-1.47)
23 wk 0 d to 26 wk 6 d	27/45 (78)	25/43 (84)	0.92 (0.39-2.16)
Death	11/45 (24)	7/43 (16)	0.60 (0.20-1.73)
Grade 3-4 IVH	11/45 (24)	7/43 (16)	0.60 (0.20-1.73)
BPD	16/45 (35)	17/43 (39)	1.18 (0.50-2.81)
27 wk 0 d to 29 wk 6 d	12/59 (20)	10/62 (16)	0.75 (0.29-1.90)
Death	2/59 (3)	2/62 (3)	0.95 (0.12-6.97)
Grade 3-4 IVH	1/59 (2)	1/62 (2)	0.95 (0.05-15.55)
BPD	9/59 (15)	7/62 (11)	0.70 (0.24-2.04)
Vaginal delivery	15/40 (38)	18/51 (35)	0.76 (0.35-1.64)
Death	5/40 (13)	4/51 (8)	0.59 (0.15-2.38)
Grade 3-4 IVH	6/40 (15)	7/51 (14)	0.90 (0.27-2.93)
BPD	9/40 (22)	11/51 (21)	0.95 (0.41-2.20)
Cesarean delivery	24/64 (38)	17/54 (31)	0.91 (0.38-2.14)
Death	8/64 (13)	5/54 (9)	0.71 (0.21-2.32)
Grade 3-4 IVH	6/64 (9)	1/54 (2)	0.18 (0.02-1.54)
BPD	16/64 (25)	13/54 (24)	0.94 (0.34-2.57)

Abbreviations: BPD, bronchopulmonary dysplasia; IVH, intraventricular hemorrhage; OR, odds ratio; PCI, placental circulation intact; UCM, umbilical cord milking.

**Table 3.** Intention to Treat Analysis of Grade 3-4 IVH, Death, and BPD

Outcome	Newborns, No. (%)		OR (95% CI)
	UCM (n = 104)	PCI (n = 105)	
Grade 3-4 IVH	12 (12)	8 (8)	0.63 (0.24-1.61)
Death	13 (13)	9 (9)	0.65 (0.26-1.60)
BPD	25 (24)	24 (23)	0.93 (0.49-1.77)

Abbreviations: BPD, bronchopulmonary dysplasia; IVH, intraventricular hemorrhage; OR, odds ratio; PCI, placental circulation intact; UCM, umbilical cord milking.

**Table 4.** Secondary Outcomes by Treatment Group for Intention to Treat Analysis

Outcome	Newborns, No. (%)		*P* value
	UCM (n = 104)	PCI (n = 105)	
Peak hemoglobin in first 24 h of life, median (IQR), g/dL	17.6 (16-20.5)	18.3 (15.9-20.2)	.42
Peak hematocrit in first 24 h of life, median (IQR), %	51 (46-58)	51 (46-60)	.38
Needed blood transfusion	76 (73)	75 (71)	.70
No. of blood transfusions, median (IQR)	2 (0-4)	2 (0-3)	.61
Mechanical ventilation in first 24 h of life	36 (35)	45 (43)	.22
Noninvasive ventilation	93 (89)	99 (94)	.19
Mechanical ventilation	49 (47)	60 (57)	.14
Inhaled nitric oxide therapy	16 (15)	19 (18)	.59
Patent ductus arteriosus treatment	51 (49)	53 (50)	.83
Length of stay, median (IQR), d	81 (53-100)	76 (55-102)	.90
Peak serum bilirubin, median (IQR), mg/dL	8.7 (6.7-10.0)	8.5 (7.0-9.8)	.46
Necrotizing enterocolitis	2 (2)	2 (2)	.99
Periventricular leukomalacia	4 (4)	2 (2)	.40
Intraventricular hemorrhage grade 1 and 2	14 (13)	18 (17)	.71
Retinopathy of prematurity	28 (27)	21 (20)	.23
Early-onset sepsis	8 (8)	3 (3)	.14
Late-onset sepsis	36 (35)	30 (28)	.11

Abbreviations: PCI, placental circulation intact; UCM, umbilical cord milking.

SI conversion factors: To convert bilirubin to micromoles per liter, multiply by 17.104; hemoglobin to grams per liter, multiply by 10; hematocrit to proportion of 1.0, multiply by 0.01.

## Discussion

To our knowledge, this is the first randomized clinical trial comparing the effects of resuscitation at birth with PCI for 180 seconds with resuscitation with UCM on outcomes of preterm newborns born at less than 30 weeks’ gestation. We found that the primary composite outcome of death, grade 3 to 4 IVH, and BPD were not significantly different between the groups.

In detail, the incidence of death was 9% in the PCI group and 13% in the UCM group. This finding is in agreement with 2 recent meta-analyses^3,32^ that reported similar mortality in newborns resuscitated with delayed cord clamping (any timing) or UCM. Moreover, in a large study comparing a medium delay (60 seconds) of cord clamping with UCM in newborns born at less than 32 weeks’ gestation, Katheria et al^33^ found no difference in death rate between the groups (6% and 7%, respectively). Current evidence agrees that delayed cord clamping in preterm newborns, especially if prolonged (≥120 seconds),^34^ is not associated with less or more mortality than UCM. It is noteworthy that the PP analysis of our data showed a reduction (from 16% to 7%) in the incidence of death in the subgroup of newborns born at 23 to 26 weeks of gestation in PCI and not in the UCM group (eTable 1 in Supplement 2).

In our trial, the incidence of grade 3 to 4 IVH was 8% in the PCI group and 12% in the UCM group. This result is in disagreement with the meta-analysis by Seidler et al,^32^ who found that delayed cord clamping (any timing) was associated with significantly less incidence of grade 3 to 4 IVH compared with UCM in newborns born before 32 weeks of gestation, but their finding was related to events in newborns born at less than 28 weeks of gestation. Katheria et al^33^ found that in newborns born at 23 to 27 weeks of gestation, grade 3 to 4 IVH was significantly reduced in the medium delayed cord clamping group in comparison with the UCM group (6% vs 22%), but this finding was not confirmed in the subgroup of less immature newborns born at 28 to 32 weeks gestation.^18^ On the basis of the results of the trial by Katheria et al,^33^ resuscitation guidelines recommended against UCM in preterm newborns born at less than 28 weeks owing to a potentially increased risk of IVH.^4,6^ In our trial, we found that in newborns born at 23 to 26 weeks of gestation, the occurrence of grade 3 to 4 IVH was similar in PCI compared with the UCM group (16% vs 24%). It should be noted that grade 3 to 4 IVH was more frequent in our PCI group than in the medium delayed cord clamping group in more immature newborns studied by Katheria et al^33^ (16% vs 6%), suggesting that PCI might be less protective than 60-second delayed cord clamping. The PP analysis of our data showed a reduction (from 16% to 10%) of the incidence of 3 to 4 IVH in the subgroup of newborns born at 23 to 26 weeks of gestation in PCI and not in the UCM group (eTable 1 in Supplement 2), suggesting that these different results might be due to the lower adherence to protocol in our than in the study by Katheria et al^33^ (69% vs 90%, respectively). Twenty percent of newborns in the PCI group received early cord clamping, which may have increased the rates of grade 3 to 4 IVH in the intervention group. In our trial, a short umbilical cord and placenta abruption were the most frequent reasons for not performing PCI at birth. To perform PCI in the presence of a short umbilical cord, the resuscitation trolley must be positioned very near the mother, and this was easier for vaginal than for cesarean births.

We found that the incidence of BPD was 23% and 24% in the PCI and UCM groups, respectively. These results confirm previous findings that delayed cord clamping (any timing or 60-second delay) and UCM were associated with a similar risk of developing BPD.^3,32,33^

Our study did not confirm the hypothesis that a long delay in clamping the cord allows smoother cardiorespiratory adaptation compared with UCM in preterm newborns and improves their outcome. On the contrary, our findings suggest that resuscitation with PCI and UCM probably provides a comparable placental transfusion and cardiorespiratory adaptation at birth. Most of the evidence indicates that ventilating the lungs before clamping is crucial to avoid harmful hemodynamic fluctuations at birth. However, it is not known how long the placental circulation should be maintained intact as the newborn begins to breathe spontaneously or with respiratory support. Possible explanations of our results might be that (1) the majority of patients in the UCM group may have started breathing during UCM, thus limiting the disadvantage of the immediate cord clamping, and (2) not all newborns benefited from a 3-minute initial ventilation period before delayed cord clamping.

It has been demonstrated in preterm lambs that asphyxia, with bradycardia and decrease in left and right ventricular outputs, does not occur immediately after cord clamping but when the interval between cord clamping and the start of spontaneous breathing or ventilatory support is longer than 40 seconds.^35^ Moreover, the negative hemodynamic effects of UCM, described as similar to those caused by immediate cord clamping, but repeated at each milk of the cord,^36^ are significantly reduced in preterm lambs whose lungs are being ventilated before and during UCM.^37,38^ Therefore, UCM should probably not be performed immediately after birth for newborns who are still not breathing, but only while the newborn is breathing (spontaneously or induced by tactile stimulation) or is being ventilated, to minimize its potential negative hemodynamic effects. Unfortunately, we were not able to collect the times of onset of spontaneous breathing in our patients, but all newborns were stimulated before clamping, including those in the UCM group during the milking procedure. This issue should be investigated in future trials.

An extended period of initial ventilation before delayed cord clamping (at approximately 5 minutes) determined a greater degree of left-to-right shunt across the ductus arteriosus with a substantial increase of pulmonary flow at the expense of flow in the aorta in preterm lambs.^39^ These hemodynamic effects might also occur in some preterm newborns, thus resulting in no beneficial effect of prolonging ventilation before delayed cord clamping. Resuscitation with an intact cord for 2 minutes did not improve the composite outcome (IVH or death rate) compared with delayed cord clamping for 30 to 60 seconds in newborns born at less than 29 weeks of gestation.^40^ It is noteworthy that grade 3 to 4 IVH was less frequent in the intervention group than in the control group (9% vs 15%) among newborns not breathing well at 30 seconds after birth,^40^ suggesting that resuscitation with an intact cord for 2 minutes might be more protective only for newborns who are not vigorous at birth.

### Limitations

This trial has some limitations. We failed to record whether the newborn was breathing at birth, which would have provided further information about why the rates of outcomes did not differ between groups. Approximately 30% of the PCI newborns were not able to follow the protocol, in the majority of cases because a short umbilical cord did not allow the newborn to be well positioned on the trolley for safe resuscitation.

## Conclusions

Compared with UCM, resuscitating preterm neonates with a PCI for 180 seconds did not improve the composite outcome of death, grade 3 to 4 IVH, and BPD. Further studies are necessary to evaluate the benefit of resuscitation with PCI in preterm newborns not breathing at birth.

## Notes

Supplement 1.
Trial Protocol and Statistical Analysis Plan

Supplement 2. **eTable 1.** Primary composite outcome overall and in prespecified subgroups (per protocol), rate and (%) **eTable 2.** Per protocol analysis of grade 3-4 IVH, death, and bronchopulmonary dysplasia, rate and (%) **eTable 3.** Secondary outcomes by treatment group (per protocol)

Supplement 3.
PCI Trial Collaborators

Supplement 4.
Data Sharing Statement
