# Supplementary material for: Resuscitation With Placental Circulation Intact Compared With Cord Milking: A Randomized Clinical Trial
Source: JAMA Netw Open. 2024 Dec 13;7(12):e2450476. doi: 10.1001/jamanetworkopen.2024.50476 (PMC11645650; doi:10.1001/jamanetworkopen.2024.50476)
Supplement: Supplement 1. — Trial Protocol and Statistical Analysis Plan [file jamanetwopen-e2450476-s001.pdf]

Placental circulation intact trial (PCI-T) - resuscitation with the placental circulation intact versus cord milking for very preterm newborns: study protocol and statistical analysis plan for a randomized controlled trial

Abstract

Background

Preterm newborns receiving placental transfusion at birth have better neonatal outcomes, particularly a decrease of hospital mortality. Placental transfusion strategies performed in preterm babies at delivery have been delayed cord clamping and cord milking. However, both these strategies did not explore the contribution of the start of breathing during placental transfusion, because they are performed in a short time frame (30-60 sec for delayed clamping and <20 sec for cord milking). Thus, newborn bedside assistance near to the delivering mother, leaving the cord unclamped, would allow to evaluate the possible beneficial effects of prolonged transfusion strategy associated to newborns' breathing, both spontaneously started or artificially supported during initial steps of stabilization, on early infant postnatal adaptation and outcomes.

Study Design

We present the design of a randomized, multicenter, controlled study, whose aim is to assess the effectiveness of delivery room assistance with intact placental circulation in comparison to cord milking for improving outcomes in very preterm newborns. A total of 212 infants born at 23+0 to 29+6 weeks of gestational age will be enrolled and randomized to receive assistance with intact placental circulation or cord milking at birth.

The primary outcome of the study is the comparison between groups of the risk of developing the composite outcome of grade 3-4 intraventricular hemorrhage (3-4 IVH), bronchopulmonary dysplasia (BPD) and mortality. Secondary outcomes include the comparison between groups of the need and duration of respiratory support, hemodynamic parameters and cerebral oxygenation, need and number of blood transfusions and inotropic drugs use in the first 24 hours.

Discussion

The results of this study will provide new information about the effectiveness of assistance with the placental circulation intact for improving outcomes in the neonatal period of preterm babies. Delaying cord clamping at 3 minutes of life while starting ventilation assistance (if needed) of the baby could permit to evaluate the contribution of the start of an efficacious breathing (spontaneous or assisted) before clamping on the early postnatal adaptation and outcomes in the neonatal period.

Keywords: randomised trial, preterm birth, delayed cord clamping, neonatal care at the bedside, cord milking, neonatal resuscitation

Trial Registration: Clinicaltrials,gov NCT02671305 (date of registration: 26 JAN 2016)

https://clinicaltrials.gov/show/NCT02671305

INTRODUCTION

Leaving the cord unclamped during the first minutes of life determines a net passage of 25 to 40 ml/kg of blood from the placenta to the newborn depending on gestational age, timing of cord clamping, postnatal infant position, onset of breathing, and administration of uterotonics to the mother (1). Placental transfusion (PT) seems to play an important role in the respiratory and hemodynamic adaptation of the newborn during the transition phase (2). In fetal life, oxygenated blood coming from the placenta reaches the heart left ventricle through the umbilical vein and foramen ovale (3). Therefore, the unphysiological immediate clamping of the cord at birth determines an abrupt and prolonged reduction of the left ventricle preload, which will be maintained till an optimal blood return from the lungs through pulmonary veins will occur. The onset of spontaneous or assisted newborn's lung ventilation increases pulmonary blood flow and venous return to left ventricle, thus restoring more or less rapidly the left ventricle preload. These early major fluctuations of the cardiac output (abruptly decreased and then increased again) are associated with fluctuations of cerebral blood flow and pressures in animals (4). Instead, a physiological-based cord clamping, that is delaying cord clamping till after the animal/newborn's breathing, has not been associated with such hemodynamic fluctuations, thus potentially preventing cerebral vasculature rupture (5) and reducing the risk of critical neonatal outcomes (6).

However, very preterm infants only briefly (30-60 sec) delayed cord clamped, as in the majority of published trials, might not receive the best benefits from the procedure, as a time period equal or less than 1 minute of life is too short to obtain an optimal ventilation of the lungs. On the basis of these considerations, we hypothesized that neonatal assistance in the delivery room during a prolonged placental circulation intact (PCI) allows a better postnatal adaptation and improves outcome in preterm infants <30 weeks gestation.

Cord milking (UCM), that is pinching the cord close to the mother and running the fingers towards the infant at a speed of 10cm/sec, usually repeated 3 to 5 times, is an unphysiological procedure able to rapidly (usually within 20 sec of life) deliver a PT at birth. However, little is known about the acute physiological effects of UCM and how it influences the cardiovascular transition after birth. UCM could potentially determine harmful hemodynamic changes similar to those occurring after immediate cord clamping at birth. To date, all published studies demonstrate that UCM may have some advantages over immediate cord clamping at birth, such as lower risk for oxygen requirement at 36 weeks and IVH of all grades, and over short delayed cord clamping, such as higher initial Hb, higher blood pressure, and improved systemic blood flow and urine output for infants delivered by cesarean section (7-8).

The present study has been designed to assess the effectiveness of assistance with PCI clamping cord at 3 min of life in comparison with UCM for improving outcome in preterm infants.

STUDY DESIGN AND POPULATION

Study design

This is a randomized, open-label, parallel-group, multicenter, prospective clinical trial, involving eight Neonatal Intensive Care Units (NICUs) in Italy. The trial was designed following the SPIRIT 2013 statement (see populated SPIRIT checklist provided as an Additional file). Patients who meet inclusion criteria will receive a bedside assistance with PCI immediately after birth and during the first 3 min of life or cord UCM (milking four times 20 cm of cord) followed by cord clamping within 20 sec of life.

Study population

The study is carried out in eight Italian NICUs. The participating centers are listed below:

- Neonatal intensive Care Unit, Division of Neonatology, Careggi University Hospital of Florence, Firenze, Italy
- Neonatal Intensive Care Unit, Division of Neonatology, Fondazione IRCCS “Ca’ Granda” University Hospital, Milano, Italy
- Neonatal Intensive Care Unit, “Maggiore” Children Hospital of Bologna, Bologna, Italy
- Neonatal Intensive Care Unit, Division of Neonatology, “Infermi” Hospital, Rimini, Italy
- Neonatal Intensive Care Unit, San Bortolo Hospital, Vicenza, Italy
- Neonatal Intensive Care Unit, Azienda Ospedaliera of Perugia, Italy
- Neonatal Intensive Care Unit, Di Venere Hospital, Bari, Italy
- Neonatal Intensive Care Unit, MBBM Foundation, San Gerardo Hospital, Monza, Italy

Infants with a gestational age between 23+0 and 29+6 weeks will be eligible for inclusion. Infants of twin pregnancies or born after placental and cord abnormalities, major congenital malformations, hydrops fetalis, and maternal severe compromise at delivery will be excluded.

Primary Outcome

The primary outcome of the study is the reduction of incidence of the composite outcome represented by 3-4 grade IVH, BPD, and mortality in preterm infants assisted at birth with the PCI in comparison with UCM. The occurrence of the composite outcome will be assessed at 36 weeks’ gestational age.

Secondary Outcomes

Secondary outcome measurements include the following:

1) 3-4 grade IVH
2) BPD
3) Mortality

Other secondary collected data will be:

1) Need for mechanical ventilation in the first 24 h of life
2) Peak hemoglobin level and hematocrit in the first 24 h of life
3) Need and number of blood transfusions
4) Need and duration of non invasive and invasive respiratory support
5) Use of inotropic drugs in the first 24 hours of life,
6) Cerebral oxygenation in the first 24 hours,
7) Superior vena cava blood flow in the first 24 hours.

Further collected data

The following data will be recorded for each infant: gestational age; birth weight; sex; mode of delivery; Apgar score at 1 and 5 min; main maternal pathologies; antenatal steroid treatment; maternal analgesia/anesthesia; highest FiO_2 in the delivery room; need for resuscitation procedures at birth (positive pressure ventilation, intubation, chest compressions, drugs); body temperature at admission; hemoglobin and hematocrit maximum values in the first 24 hours; the highest FiO_2 and mean airway pressure (MAP) values; need and duration of non-invasive (NCPAP, biphasic positive airway pressure, nasal intermittent mandatory ventilation), and invasive (patient-triggered ventilation or high frequency oscillatory ventilation) respiratory support; and need and duration of nitric oxide therapy. We will also report the occurrence of patent ductus arteriosus (PDA) requiring pharmacological or surgical treatment, of IVH, periventricular leukomalacia (PVL), NEC, ROP, and hospital stay duration.

A diagnosis of sepsis will be based on clinical and laboratory data (white cell count, C-reactive protein concentration) and will be confirmed by positive blood cultures (9). IVH will be graded according to a standard classification system (10). A diagnosis of PVL will be made when cystic areas are detected by cerebral ultrasonography at 40 weeks post-conception birth (11). BDP will be diagnosed according to the definition by Jobe and Bancalari (12). ROP will be graded according to the international classification of retinopathy of prematurity (13). NEC will be diagnosed in agreement with classical Bell’s criteria (14).

Sample Size Calculation

The frequency of the composite primary outcome in the participating Italian neonatal units is about 47% (severe IVH 8.0%, BPD 24,4%, mortality 14,6%). Meta-analyses showed a reduction of 32% in in-hospital mortality and of 17% in any IVH in preterm infants assisted at birth with delayed cord

clamping instead of immediate cord clamping (6;24). Moreover, no data on the incidence of outcomes between UCM and delayed cord clamping is available. Thus, we arbitrarily hypothesized that neonatal assistance with PCI might decrease the frequency of the composite primary outcome from 47% to 28%. Considering a dropout rate of 5%, we calculated that 106 newborns must be enrolled in each group to detect this difference as statistically significant with 80% power at a level of 0.05 using a 2-sided chi-square test.

TREATMENT OF SUBJECTS

Randomization

Infants at each unit are randomly assigned in blocks to a treatment group (PCI or UCM) in a 1:1 ratio. The allocation sequences consist of computer generated random numbers. Since the frequency of the composite primary outcome is inversely related to gestational age, the inclusion of patients is balanced in each treatment group according to the following gestational ages: 23+0 to 26+6 weeks or 27+0 to 29+6 weeks.

Allocation concealment

The enrolled patients are allocated to the treatment arm using an electronic randomization system. This is a secure web-based randomisation system which could be accessed from a desktop computer.

Patient enrollment and allocation to treatment are the responsibility of neonatologists involved in the study at participating centers.

If a baby allocated to PCI procedure will not undergo it, investigators will highlight the timing and reason for an early cord clamping.

Blinding

The study is not blinded and the staff performing the study also care for the infants. However, the cardiac and cerebral echografic assessments are made by clinicians other than investigators involved in patient care, and researchers assessing study endpoints are blinded to the study treatments.

Study Procedures

Patients are randomly allocated to one of the following treatment groups:

– Group I: patients are assisted with the PCI while lying supine on a portable resuscitation trolley (Lifestart trolley™, Inspiration Healthcare, UK) and the cord is clamped at the end of the 3rd min of life. In case the treatment is not performed due to maternal or neonatal reasons the infant will receive an immediate cord clamping.

– Group II: patients receive cord UCM without placental refill (milking four times 20 cm of cord at a speed of about 10 cm/sec) and then clamped before 20 sec of life. In case the treatment is not

performed due to maternal or neonatal reasons the infant will receive an immediate cord clamping.

Compliance will be defined as full adherence to the protocol. Compliance with the protocol will be ensured by a number of procedures included in the site set-up. The local principal investigator participated in preparatory meetings in which details of the study protocol and data collection were accurately discussed. All centers received detailed instruction on study procedures and web-based recording data.

All infants will be resuscitated following the current guidelines of the American Academy of Pediatrics (AAP) (15). Neonatal care will be started with FiO_2 of 0.30 in both the groups, in agreement with local protocols. Respiratory support in the delivery room will be given using a T-piece ventilator (Neopuff Infant T-Piece Resuscitator, Fisher & Paykel, Auckland, New Zealand), which is a pressure-limited mechanical device that supplies consistent peak inspiratory pressure (PIP) and positive end expiratory pressure (PEEP), and can deliver sustained inflation (16). Initial PIP and PEEP values will be set at 20 and 5 cmH_2O respectively. To avoid pressure leakage, a neonatal mask of appropriate size, which adequately covers both the mouth and nostrils of infants, will be used. The flow rate will be set at 10 L/min without changes during the resuscitation. Spontaneously breathing babies with a respiratory effort present or with a suboptimal SpO_2 on pulse oximeter will be assisted in nCPAP. In the delivery room, infants will be started on mechanical ventilation (MV) if they have not reached the goal of 70% SpO_2 by 5 minutes (17) and 85% by 10 minutes of life (18) with a heart rate >100 bpm, despite nasal CPAP at 5-8 cmH_2O.

A pulse oximeter (Radical-7, Masimo Corporation, USA) is positioned on Lifestart trolley, and the trolley, together with all devices placed on it, is covered with a transparent sterile coverage in case of cesarean section. Thus, the obstetrician hands place the newborn on a completely sterile trolley and then come back to the mother abdomen, at that moment the neonatologist begin to assist the baby (with aspiration if necessary, positioning pulse oximeter probe, starting nCPAP/PPV as necessary). Heart rate will be assessed by auscultation and pulse oximeter. For babies assisted with an intact cord on Lifestart trolley two person provide bedside neonatal assistance (1 doctor and 1 nurse). During the 3-minutes period of delayed cord clamping the prevalent procedure will be to assist respiratory function with nCPAP/PPV till intubation if necessary. If necessary, an early surfactant administration will be performed in delivery room, but not during the 3-minutes period of delayed cord clamping.

Daily clinical care of the enrolled patients will be performed by the attending physicians in accordance with the common practice at each center. Daily fluid intake will be started at 80-90 ml/kg and gradually increased by 10–20 ml/kg/day based on changes in body weight, serum sodium concentrations, and osmolality, with a target intake of 150–160 ml/kg at the end of the first week of life. In case of hypotension refractory to the fluid-replacement therapy, dopamine

and/or dobutamine treatment will be provided. For the treatment of respiratory distress syndrome (RDS), infants will receive oxygen therapy, respiratory support, and rescue surfactant treatment to achieve the following therapeutic targets: PaO_2 50–60 mmHg, $PaCO_2$ < 65 mmHg, pH> 7.20, and SpO_2 90–95 %. To maintain an adequate SpO_2, infants with an FiO_2 ≥0.30 will be treated with surfactant (200 mg/kg Curosurf, Chiesi Pharmaceuticals, Parma, Italy) through the intubation-surfactant-extubation (INSURE) procedure or less invasive surfactant administration (LISA) strategy. All infants who will need MV will be treated with surfactant and will then be gradually weaned from it. Additional doses of surfactant (100 mg/kg) will be given to infants, also through the INSURE strategy, at the discretion of the attending neonatologist. Infants will be started on MV when the pH is <7.20 with $PaCO_2$ >65 mmHg, PaO_2 <50 mmHg with FiO_2 ≥0.50 after surfactant treatment, or if infants have frequent episodes of apnea (>4 episodes in 1 hour or >2 episodes requiring bag-and-mask ventilation), despite adequate nasal CPAP (5-8 cmH_2O) delivery and oxygenation. MV will be set to maintain a $PaCO_2$ of 55-65 mmHg and SpO_2 of 88-95%. Infants will be extubated when they meet all of the following criteria: FiO_2<0.40, $PaCO_2$<65 mmHg with a pH >7.20, MAP ≤7 cmH_2O with hemodynamic stability.

Prophylactic antibiotics will be administered from the time of admission to the NICU and will be stopped after 3 days if the results of the bacterial cultures remain negative.

All newborns will receive continuous cerebral oxygenation monitoring by near infrared spectroscopy (NIRS), and echographic assessments of cardiac function, and cerebral and mesenteric blood flow parameters in the first 24 hours of life.

Participant timeline

The patients enrolled in the trial do not receive extra laboratory tests. A heart ultrasound is performed in the first 24 hours of life and then repeated until PDA closure or as needed. The direction of ductal shunt, right and left ventricular stroke volume, and superior vena cava flow are recorded at the first heart ultrasound. All echographic studies are performed in participating centers by pediatric cardiologists or by neonatologists trained and certified for neonatal echocardiography, fulfilling the current recommendations for echocardiography training in the NICU (19).

Serial cerebral ultrasounds for evidence of possible IVH and/or PVL are performed in the first 24 hours of life, at 2, 3, 6, 7 (±1), 15 (±2), and 30 (±2) days of life, and at 40 weeks post-conception. We will collect severe IVH but also Grade 1 and 2 IVH. We will consider only local reads. Cerebral oxygenation monitoring by near infrared spectroscopy is performed as soon as possible after the admission of the newborn in NICU and last 24 hours.

PROCEDURES

Recruitment and Consent

Women at risk of very preterm birth will be offered participation, and if they accept will give written consent. Investigators strictly monitor everyday admissions in obstetrics ward and give early consent to every eligible woman in order to minimize the risk of missing enrolment due to quickly delivery. Before patients are entered into the study, the parents (or legal representatives) will be fully informed about the purposes of the study, possible benefits, any potential reasonable risk or discomfort, the expected duration of the newborn's participation, the clinical and/or functional procedures, as well as the name of the investigator(s) responsible for conducting the trial and his/her direct contact details. Written and oral information will be offered to parents, and sufficient time will be allowed for consent. The newborn will enter the study only after both parents or legal representatives sign the informed consent form. Eligibility and consent will be checked before randomization, which will be during labour or before caesarean section.

Whenever antenatal consent is obtained, but the mother passes the term of 30 weeks of gestation, the infant will not be included in this study.

Non-Italian speaking parents will only be asked for their consent if an adult interpreter is available. Trust interpreter and link worker services will be used to support involvement of participants whose first language is not Italian. A senior investigator will be always available to discuss concerns raised by parents or clinicians during the trial.

A monthly accrual report about the study is sent to participating centers.

Data Safety and Monitoring Board

Safety and tolerability will be assessed by monitoring the frequency and severity of adverse events. Safety end-points will include incidence, severity and causality of reported serious adverse events (SAEs), namely changes in occurrence of the expected common prematurity complications and routine clinical laboratory test assessments, and the development of unexpected SAEs in this high risk population. All SAEs will be followed until satisfactory resolution or until the investigator responsible for the care of the participant deems the event to be chronic or the patient to be stable. All expected and unexpected SAEs will be reviewed by the local principal investigators to determine if there is a reasonable suspected causal relationship with the intervention. A monitoring board including an independent assessor (not involved in the study) from the IRCCS San Martino Hospital (Genova, Italy) and assessors from each participating hospital will review all the deaths and adverse effects. If there is a reasonable suspected causal relationship with the intervention, SAEs will be reported to the Ethics Committee to guarantee the safety of the participants.

An interim analysis will be performed on the primary endpoint and SAEs from the first 100 infants enrolled. The interim analysis will be performed by the statistician, blinded for the treatment allocation, who will report to the principal investigator. The principal investigator will discuss the results of the interim analysis with the monitoring board and the trial will be ended in case of

harm. Criteria for stopping for harm include: 1) a statistically significant difference in SAEs between the treatment groups, and 2) a reasonable suspected causal relationship between the intervention and SAEs, and 3) a significant increase in occurrence of SAEs in respect to the incidence reported in newborns of same gestational age in Vermont Oxford Database.

Statistical Analysis

The primary efficacy analysis will be conducted on an intention-to-treat basis and the per-protocol approach for a secondary sensibility analysis. Clinical characteristics of infants in PCI and UCM groups will be described using mean values and standard deviation, median value and range, or frequencies and percentage.

Univariate statistical analysis will be performed using the Wilcoxon rank-sum test for continuous variables and the chi-square test or Fisher's exact test when appropriate for categorical variables. A two-sided P value <0.05 will be considered statistically significant.

Relative effect estimates will be expressed as odds ratio (OR) with Wald 95% confidence limits.

With explorative intent we will evaluate the hetereogeneity of the results within the two groups of newborns stratified by the type of delivery.

Statistical analysis will be performed with SAS System version 9.4 (SAS Institute Inc.).

Data collection methods

All collected data will be obtained from the clinical records. Data collection forms are included in the electronic case report form (eCRF) that was specifically designed for the trial using a not validated web-based proprietary application that is compliant with the European regulations regarding electronic records and signatures. Subjects are identified by sex, birth date, and assigned trial number only, in accordance with personal data protection law. The study coordinator and trial statistician will review the generated results for logic, coherence, or problems. Outlier data will be investigated.

Once an infant is enrolled or randomized, the study site will make every reasonable effort to follow the infant for the entire study period.

Ethics and Dissemination

The study protocol has been carried out in accordance with the principles of the Declaration of Helsinki and approved by the following local ethical committees of the participating centers: the Pediatric Ethical Committee of Tuscany (Firenze), Ethical Committee of Milan-B Area (Milano), Ethical Committee of Bologna (Bologna), Ethical Committee of San Bortolo Hospital (Vicenza), Ethical Committee of Romagna (Rimini), Ethical Committee of Umbria (Perugia), Ethical Committee of Puglia (Bari), Ethical Committee of San Gerardo Hospital (Monza).

Possible protocol amendment during the study period will be submitted for approval to all the aforementioned ethical committees.

The study coordinator and trial statistician will have full access to the complete final dataset. Site investigators will have direct access to their own site's data sets, and will have access to other sites data by request, only after completion of the data analysis. The datasets during and/or analysed during the current study will be available from the corresponding author on reasonable request.

The results of the trial are expected to be published in a scientific journal and to be presented in medical seminars and conferences. The final reporting will follow the CONSORT Report guidelines (http://www.consort-statement.org).

DISCUSSION

PT strategies such as a short delay in cord clamping (30-60 sec) and cord UCM (with cord clamping within 20 sec of life) have been demonstrated to improve outcomes in preterm newborns (20,21).

It has been demonstrated that to perform initial neonatal resuscitation with PCI at mother bedside is feasible, safe and permits to obtain an effective lung ventilation before cord clamping (22,23). Most preterm infants assisted at mother bedside with their cord intact start breathing spontaneously in the first minute of life (23). Newborns who start breathing or ventilating before cord clamping have better outcomes than newborns who start breathing or ventilating after cord clamping (24). In the ongoing UK Cord Trial preterm infants <32 weeks gestation randomized in the experimental arm receive cord clamping after at least 2 min of life and immediate neonatal care is given with intact cord, while the control arm receive an immediate cord clamping at birth. Our choice of performing cord clamping after 3 min of life in the study group is functional to leave the establishing of an effective ventilation of the lungs before clamping. But, it will be important to obtain a high adherence to the protocol in the experimental study group. The more physiological approach of stimulating spontaneous breathing or starting ventilation if necessary during the first 180 sec of life before clamping the cord of very preterm newborns might result neuroprotective, reducing the intraventricular hemorrhage rate compared with cord milking. To date, there is evidence that cord UCM at birth has some benefits versus immediate cord clamping (7,8), so the priority is now to compare cord milking with delayed clamping (25), but the delay should be long enough to really consider the procedure a physiological-based cord clamping (26).

Thus, we hypothesized that clamping the cord after 3 min of life might be more beneficial than cord UCM in preterm infants <30 weeks gestation because during this period neonates can start to breathe/ventilate having the aforementioned hemodynamic advantage and a more physiological postnatal transition phase.

LIST OF ABBREVIATIONS

UCM: milking; PCI: placental circulation intact; PT: placental transfusion; eCRF: electronic case report form; IVH: intraventricular hemorrhage; MV: mechanical ventilation; NCPAP: nasal continuous airway pressure; NEC: necrotizing enterocolitis; NICU: neonatal intensive care units; PVL: periventricular leukomalacia; ROP: retinopathy of prematurity.

COMPETING INTERESTS

There no competing interest to declare.

FUNDING

The authors declare that they have no funding to declare.

AUTHORS' CONTRIBUTIONS

Simone Pratesi conceived the protocol and contributed to the protocol development and eCRF improvement. Simone Pratesi wrote the first draft of the protocol. Carlo Dani contributed to the protocol development and eCRF improvement. Stefano Ghirardello contributed to interpretation of the study protocol in relation to other studies performed on this subject. Luca Boni prepared electronic data sheets and statistical analysis plan, and was responsible for the web-based electronic case record form. Fabio Mosca, Fabrizio Sandri, Giovanna Mescoli, Roberta Corbetta, Miria Natile, Stefania Vedovato, Stefania Troiani, Cristiana Germini, and Flavia Petrillo contributed to the protocol revision. All authors read and approved the final version of the protocol.

COORDINATING CENTER

The NICU of Careggi University Hospital of Florence is the coordinating center of the study and was the principal developer of the study protocol. During the study period, the coordinator center and the other participating centers will equally contribute to patient enrollment and data collection although differences in the number of enrolled patients per center are expected.

REFERENCES

1. Van Rheenen PF, Brabin BJ. A practical approach to timing cord clamping in resource poor settings. *BMJ* 2006,333:954-958. DOI: 10.1136/bmj.39002.389236.BE
2. Bhatt S, Alison BJ, Wallace EM, Crossley KJ, Gill AW, Kluckow M, et al. Delaying cord clamping until ventilation onset improves cardiovascular function at birth in preterm lambs. *J Physiol* 2013,591(8):2113-26. DOI: 10.1113/jphysiol.2012.250084

3. Crossley KJ, Allison BJ, Polglase GR, Morley CJ, Davis PG, Hooper SB. Dynamic changes in the direction of blood flow through the ductus arteriosus at birth. *J Physiol* 2009, 587(19):4695-704. DOI: 10.1113/jphysiol.2009.174870
4. Hooper SB, Te Pas AB, Lang J, van Vonderen JJ, Roehr CC, Kluckow M, et al. Cardiovascular transition at birth: a physiological sequence. *Pediatr Res* 2015, 77(05):608–614. DOI: 10.1038/pr.2015.21
5. Bhatt S, Polglase GR, Wallace EM, Te Pas AB, Hooper SB. Ventilation before umbilical cord clamping improves the physiological transition at birth. *Front Pediatr* 2014, 2:113. DOI: 10.3389/fped.2014.00113
6. Rabe H, Diaz-Rossello JL, Duley L, Dowswell T. Effect of timing of umbilical cord clamping and other strategies to influence placental transfusion at preterm birth on maternal and infant outcomes. *Cochrane Database Syst Rev* 2012, (8):CD003248. DOI: 10.1002/14651858.CD003248.pub3
7. Hosono S, Mugishima H, Fujita H, Hosono A, Okada T, Takahashi S, et al. Blood pressure and urine output during the first 120 h of life in infants born at less than 29 weeks' gestation related to umbilical cord milking. *Arch Dis Child Fetal Neonatal Ed* 2009, 94(5):F328-31. DOI: 10.1136/adc.2008.142935
8. Al-Wassia H, Shah PS. Efficacy and safety of umbilical cord milking at birth. A systematic review and meta-analysis. *JAMA Pediatr* 2015, 169: 18-25. DOI: 10.1001/jamapediatrics.2014.1906
9. Goldstein B, Giroir B, Randolph A. International pediatric sepsis consensus conference: definitions for sepsis and organ dysfunction in pediatrics. *Pediatr Crit Care Med* 2005, 6:2–8. DOI: 10.1097/01.PCC.0000149131.72248.E6
10. Papile LS, Burstein J, Burstein R, Koffler H. Incidence and evolution of the subependymal intraventricular hemorrhage: a study of infants weighing less than 1500 grams. *J Pediatr* 1978, 92:529–34. DOI: 10.1016/s0022-3476(78)80282-0
11. de Vries LS, Eken P, Dubowitz LM. The spectrum of leukomalacia using cranial ultrasound. *Behav Brain Res* 1992, 49:1–6. DOI: 10.1016/s0166-4328(05)80189-5
12. Jobe A, Bancalari E: Bronchopulmonary dysplasia. Am J Respir Crit Care Med 2001, 163:1723–1729.
13. International Committee for the Classification of Retinopathy of Prematurity. The International Classification of Retinopathy of Prematurity revisited. *Arch Ophthalmol* 2005, 123:991–9. DOI: 10.1001/archopht.123.7.991
14. Bell MJ, Ternberg JL, Feigin RD, Keating JP, Marshall R, Barton L, et al. Neonatal necrotizing enterocolitis: therapeutic decisions based on clinical staging. *Ann Surg* 1978, 187:1–7. DOI: 10.1097/00000658-197801000-00001

15. Perlman JM, Wyllie J, Kattwinkel J, Wyckoff MH, Aziz K, Guinsburg R, et al. Neonatal Resuscitation Chapter Collaborators. Part 7: Neonatal Resuscitation: 2015 International Consensus on Cardiopulmonary Resuscitation and Emergency Cardiovascular Care Science With Treatment Recommendations. *Circulation* 2015, 132(16 Suppl 1):S204-41. DOI: 10.1161/CIR.0000000000000276
16. Te Pas AB, Walther FJ. A randomized, controlled trial of delivery-room respiratory management in very preterm infants. *Pediatrics* 2007, 120:322–329. DOI: 10.1542/peds.2007-0114
17. Finer N, Leone T. Oxygen saturation monitoring for the preterm infant: the evidence basis for current practice. *Pediatr Res* 2009, 65:375–380. DOI: 10.1203/PDR.0b013e318199386a
18. Vento M, Cheung PY, Aguar M. The first golden minutes of the extremely low-gestational-age neonate: a gentle approach. *Neonatology* 2009, 95:286–298. DOI: 10.1159/000178770
19. de Boode WP, Singh Y, Gupta S, Austin T, Bohlin K, Dempsey E, et al. Recommendations for neonatologist performed echocardiography in Europe: Consensus Statement endorsed by European Society for Paediatric Research (ESPR) and European Society for Neonatology (ESN). *Pediatr Res* 2016, 80(4):465-71. DOI: 10.1038/pr.2016.126
20. National Institute for Health and Care Excellence. Preterm labour and birth. *NICE Guideline*, London 2015.
21. Backes CH, Rivera BK, Haque U, Bridge JA, Smith CV, Hutchon DJ, et al. Placental transfusion strategies in very preterm neonates: a systematic review and meta-analysis. *Obstet Gynecol* 2014, 124: 47-56. DOI: 10.1097/AOG.0000000000000324
22. Thomas MR, Yoxall CW, Weeks AD, Duley L. Providing newborn resuscitation at the mother's bedside: assessing the safety, usability and acceptability of a mobile trolley. *BMC Pediatrics* 2014, 14:135. DOI: 10.1186/1471-2431-14-135
23. Katheria A, Poeltler D, Durham J, Steen J, Rich W, Arnell K, et al. Neonatal Resuscitation with an Intact Cord: A Randomized Clinical Trial. *J Pediatr* 2016, 178:75-80. DOI: 10.1016/j.jpeds.2016.07.053
24. Nevill E, Meyer MP. Effect of delayed cord clamping (DCC) on breathing and transition at birth in very preterm infants. *Early Hum Dev* 2015, 91: 407-11. DOI: 10.1016/j.earlhumdev.2015.04.013
25. Katheria AC, Truong G, Cousins L, Oshiro B, Finer NN. Umbilical cord milking versus delayed cord clamping in preterm infants. Pediatrics 2015;136:61–9.
26. Hooper SB, Binder-Heschl C, Polglase GR, Gill AW, Kluckow M, Wallace EM, et al. The timing of umbilical cord clamping at birth: physiological considerations. *Matern Health Neonatol Perinatol* 2016, 13;2:4. DOI: 10.1186/s40748-016-0032-y

STATISTICAL ANALYSIS PLAN

Delivery room assistance with the placental circulation intact: effects on early postnatal adaptation and outcome of preterm babies. Study protocol for a randomized controlled trial. The PCIT1 Trial

University of Florence

Version: 1.0

Author: L. Boni, MD
(Unit of Clinical Epidemiology, IRCCS Ospedale Policlinico San Martino, Genoa, Italy)

Date: 15-March-2023

STATISTICAL ANALYSIS PLAN APPROVAL

**Name & Title of person submitting form:** (Trial Statistician)	Luca Boni, MD
**Date Submitted:** (dd-mmm-yyyy)	15-March-2023
☒ SAP approval	☐ Amendment of SAP approval

NOTE:

1) Amendments made prior to unblinding for blinded studies or database release for open labelled studies are included in an updated SAP.

2) Amendments to the statistical analysis after unblinding or database release are documented in the Clinical Study Report.

Final Sign-off (i.e. approval) of the SAP and approval of any subsequent amendments is the responsibility of the Scientific Coordinator of the study.

The approver serves as a single point of accountability for approval and must ensure that all relevant functions agree with the final SAP.

Approvers Name: Simone Pratesi

Title: MD, PhD (Scientific Coordinator)

Signature*: [signature]

Date: 03-June-2024
(dd-mmm-yyyy)

*Signature above indicates approval of this plan, for the analyses and reporting of this trial.

This Document is Confidential.

LIST OF CHANGES			
**VERSION**	**DESCRIPTION OF CHANGES**	**AUTHOR**	**DATE**
1.0	First version.	Luca Boni	15-Mar-2023

This Document is Confidential.

LIST OF ABBREVIATIONS

ACA	Anterior cerebral artery
BPD	Bronchopulmonary dysplasia
CI	Confidence interval
ET	Endotracheal
Hb	Haemoglobin
Ht	Haematocrit
iNO	Inhaled nitric oxide
IQR	Interquartile range
i.v.	Intravenous
IVH	Intraventricular haemorrhage
ITT	Intention-to-treat
min	Minute
NEC	Necrotizing enterocolitis
PAP	Pulmonary artery pressure
PAS	Systolic blood pressure
PDA	Patent ductus arteriosus
PLV	Periventricular leukomalacia
PP	Per-protocol
RDS	Respiratory distress syndrome
ROP	Retinopathy of prematurity
SAE	Serious adverse event
SAP	Statistical analysis plan
SD	Standard deviation
SE	Standard error

This Document is Confidential.

TABLE OF CONTENTS

1 PREFACE 6

2 STUDY OVERVIEW 6

2.1 BACKGROUND 6
2.2 RATIONALE FOR PERFORMING THIS TRIAL 8
2.3 STUDY OBJECTIVES 9
2.3.1 Primary objective 9
2.3.2 Secondary objectives 9
2.4 STUDY DESIGN 9
2.5 STUDY POPULATION 9
2.5.1 Inclusion criteria 9
2.5.2 Exclusion criteria 10
2.6 INTERVENTIONAL GROUPS 10
2.7 SAMPLE SIZE 10
2.8 TREATMENT ALLOCATION 10
2.9 BLINDING AND UNBLINDING 11

3 POPULATIONS OF ANALYSIS 11

3.1 RANDOMIZED POPULATION 11
3.2 INTENTION-TO-TREAT POPULATION 11
3.3 PER-PROTOCOL POPULATION 12
3.4 SAFETY POPULATION 12
3.5 TREATMENT MISALLOCATIONS 12
3.6 PROTOCOL DEVIATIONS 12

4 ENDPOINTS AND COVARIATES 12

4.1 EFFICACY VARIABLES 12
4.1.1 Primary efficacy variable 12
4.1.2 Secondary efficacy variables 13
4.2 SAFETY VARIABLES 14
4.3 HANDLING OF MISSING VALUES 14

5 STATISTICAL AND ANALYTICAL METHODS 14

5.1 INTERIM ANALYSES, FINAL ANALYSES AND UNBLINDING 14
5.2 STATISTICAL ANALYSES 15
5.2.1 Patients' characteristics at baseline 15
5.2.2 Primary efficacy analysis 15
5.2.3 Secondary efficacy analyses 16
5.2.4 Safety Analyses 16

6 REFERENCES 16

APPENDIX 1 - PLANNED DATA DISPLAY 19

This Document is Confidential.

1 PREFACE

This Statistical Analysis Plan (SAP) is based on the version 4.0 of the trial protocol, dated December 22, 2016. The version 4.0 of the protocol amended the previous version 3.0, dated October 2, 2015, that was initially approved by the Ethical Committees and used in the first part of the trial. Previous versions were released during the development phase of the study protocol and were never adopted.

2 STUDY OVERVIEW

2.1 BACKGROUND

Placental transfusion, achieved by delaying the clamping of the umbilical cord at birth, results in better cardiorespiratory adaptation in preterm infants, an increase in mean arterial pressure and haematocrit levels in the first hours of life, improved tissue oxygenation at the cerebral and renal levels, a reduction in the number of red blood cell transfusions, a reduction in sepsis, reduced oxygen dependence, and, most importantly, a reduced incidence of cerebral haemorrhage (1, 2). Some have defined placental transfusion as the only 'drug' with proven therapeutic efficacy for the newborn in the delivery room (3).

If the umbilical cord is not immediately clamped at birth, within the first 3-5 minutes of life, there is a transfer of placental blood from the placenta to the newborn, estimated to be about 25-40ml/kg in term newborns delivered vaginally (4). In caesarean deliveries, this transfusion of blood to the newborn would occur within the first 40 seconds, after which there would be a reversal of flow favouring the placenta (5). Determinants for the occurrence of placental transfusion include uterine contractions, the patency of umbilical vessels, and the onset of effective respiration: the presence of uterine contractions (absent in caesarean delivery), vasoconstriction of the umbilical arteries but not the vein (mainly due to the increase in PaO_2 at birth), and pulmonary vasodilation following effective respiration create the pressure gradient between placental and neonatal circulation that allows placental transfusion in the first minutes of life.

Not clamping the umbilical cord at birth, if postnatal adaptation progresses normally, allows placental blood to flow from the placenta to the newborn through the umbilical vein, then into the inferior vena cava, right atrium, right ventricle, pulmonary artery, and increasingly into the pulmonary circulation as pulmonary vascular resistance decreases (and in decreasing amounts from the right atrium to the left atrium through the foramen ovale, due to the progressive increase in pressure in the left heart sections). Thus, maintaining intact placental circulation ensures left ventricular filling (and thus left ventricular output) even if pulmonary vascular resistance does not decrease in the first minutes of life due to delayed postnatal adaptation (the oxygenated blood from the placenta will pass more into the systemic circulation through the foramen ovale and ductus arteriosus, as before birth). The gradual fall in pulmonary vascular resistance will ensure that left ventricular filling (and thus left ventricular output) is increasingly maintained by pulmonary flow and less by right-to-left shunt through the foramen ovale.

This Document is Confidential.

Placental transfusion thus maintains systemic cardiac output during the respiratory adaptation phase.

The onset of effective respiration/ventilation, with the consequent lung expansion, facilitates simultaneous pulmonary vasodilation, and the increased pulmonary flow would be at least partly ensured by placental transfusion (conversely, with immediate/early cord clamping, pulmonary flow could be ensured at the expense of systemic blood 'stolen' from other organs, such as the intestines). Increased pulmonary flow in a properly ventilated lung results in “capillary erection”, which is the distention of perfused pulmonary capillaries that exerts an outward force against alveolar collapse during expiration, thus contributing to respiratory stabilization.

A recent study on preterm lambs (6) showed that clamping the cord at birth before the onset of respiration causes immediate reflex bradycardia (from average values of 160 bpm to 100 bpm) and persistent foetal circulation with a progressive reduction in right ventricular output; the subsequent improvement in pulmonary flow is then achieved by the initiation of artificial ventilation (at 2 minutes of life in the study design). Immediate cord clamping at birth also causes a sudden increase in pressure and flow in the carotid artery followed by a large reduction in flow with pressure adjustment (indicating a pressure-dependent flow in the carotid artery). In contrast, lambs ventilated for 3-4 minutes before cord clamping (until the demonstrated increase in pulmonary flow) showed, compared to those clamped immediately: 1) absence of bradycardia at the time of clamping, 2) doubled pulmonary flow, 3) stable carotid artery flow and pressure (no significant fluctuations), 4) higher pulmonary flow and right ventricular output still at 30 minutes.

Greater hemodynamic stability in the cerebral circulation in the first minutes of life could underlie the reduced incidence of cerebral haemorrhages in newborns benefiting from placental transfusion. Preterm infants undergoing delayed umbilical cord clamping have increased superior vena cava flow and a reduced incidence of severe cerebral haemorrhage (7).

The first minutes of life for preterm infants have been called “golden minutes”, as during this brief period, the newborn undergoes the most complicated phase of physiological adaptation in their lifetime, and the type of care the newborn 'receives' can significantly impact their short- and long-term future, in terms of both mortality and morbidity (8). To ensure proper and timely care for the preterm infant in the delivery room, the neonatologist must follow international neonatal resuscitation guidelines (9), applying them as “gently” as possible, quickly ensuring respiratory support that provides effective ventilation. Therefore, it is necessary to clamp and cut the umbilical cord early in infants requiring some degree of assistance/resuscitation at birth, as is the case for nearly all extremely preterm infants, to position them on a neonatal resuscitation island. The initiation of neonatal resuscitation is the main reason for the lack of delayed cord clamping in preterm infants. To achieve placental transfusion without excessively delaying the start of neonatal care, the cord milking manoeuvre (rapid squeezing of about 20 cm of umbilical cord 4-5 times from the placenta to the newborn) can be performed. Cord milking results in a placental blood transfer in about 10-15 seconds equivalent to that obtained by not clamping the cord for about 30-60 seconds, with short-term positive

This Document is Confidential.

effects similar to those achieved with delayed clamping (10, 11). Cord milking allows for partial placental transfusion but does not benefit from the positive effect that the onset of effective newborn respiration would have on enhancing the efficacy of placental transfusion itself. Artificially achieved placental transfusion with cord milking could result in reduced positive effects compared to placental transfusion occurring physiologically in a newborn already effectively spontaneously breathing or, if necessary, adequately and promptly assisted. The effect of placental transfusion on postnatal adaptation is likely maximal in the newborn adapting well spontaneously or being adequately assisted in the first minutes of life to complete the transition phase while the cord is not clamped. Some foetal-placental pathological conditions such as chronic asphyxia may also cause a redistribution of foetal-placental blood volume with an increase in foetal blood volume that, especially if associated with reduced myocardial contractility, could be aggravated by rapid induced placental transfusion via cord milking.

Initiating care for the preterm infant “with intact placental circulation”, i.e. not clamping the umbilical cord, would likely achieve the maximum benefits demonstrated by placental transfusion without delaying the start of appropriate neonatal care. The number of studies demonstrating the benefits of placental transfusion in preterm infants, achieved by delaying clamping or performing cord milking, has increased considerably in recent years (10-16), and the lack of studies on infants assisted with intact placental circulation is mainly due to the difficulty of having a suitable setting to assist the newborn next to the mother's bed (17). At the time of the design of the PCIT1 trial, a randomized controlled trial (Cord Pilot Trial) was underway in England to verify the benefits of delayed cord clamping compared to immediate clamping, assisting infants, if necessary, near the mother's bed without clamping the cord. Initial data regarding the positive evaluation by professionals and parents of the bedside setting used in the trial to assist the newborn with intact placental circulation had been published (18, 19).

2.2 RATIONALE FOR PERFORMING THIS TRIAL

Based on these premises, we hypothesized that assisting preterm infants in the delivery room without clamping the umbilical cord at birth, thereby maintaining intact placental circulation, compared to assisting them after cord milking and early clamping, would result in better postnatal adaptation. This is demonstrated in the short term by a reduction in the need for mechanical ventilation within the first 24 hours, improved cerebral and intestinal hemodynamic parameters, and reduced use of plasma expanders or vasoactive drugs, and in the medium term by a reduction in severe complications such as severe cerebral haemorrhage, bronchopulmonary dysplasia, and mortality. The decision to compare assistance with intact placental circulation to assistance after cord milking, rather than to standard preterm infant care, which still does not widely include milking, is based on the positive scientific results published in favour of the latter procedure compared to standard care (i.e., immediate cord clamping without milking in infants with a gestational age of <30 weeks).

This Document is Confidential.

2.3 STUDY OBJECTIVES

2.3.1 Primary objective

To compare in the two groups of preterm infants in which assistance with intact placental circulation or assistance after cord milking has been given the proportion of subjects experiencing at least one of the following events at 36 weeks of post-conceptional age: grade 3 or 4 intraventricular haemorrhage (IVH), moderate or severe bronchopulmonary dysplasia (BPD) or death from any cause (composite outcome).

2.3.2 Secondary objectives

- To compare in the two groups of preterm infants in which assistance with intact placental circulation or assistance after cord milking has been given the proportion of subjects experiencing at 36 weeks of post-conceptional age each of the events included in the composite outcome.
- To assess the presence of interaction between type of assistance and gestational age, and between type of assistance and type of delivery, on the primary composite endpoint and on each of its components.
- To compare in the two groups of preterm infants in which assistance with intact placental circulation or assistance after cord milking has been given the results related to the identified secondary efficacy endpoints.
- To evaluate the safety of the two assistance strategies.

2.4 STUDY DESIGN

This is a prospective, two-stage, multicentric, open-label, randomized phase III study of assistance with intact placental circulation versus assistance after cord milking in preterm infants.

In the first part of the trial, 20 preterm infants have been enrolled in the study in order to evaluate the feasibility of the experimental procedures. In the second part of the trial, the enrolment of the overall planned study population was completed. The first 20 accrued patients were included in the final statistical analysis.

2.5 STUDY POPULATION

2.5.1 Inclusion criteria

Infants who mainly met the following criteria were eligible for participation into the trial:

- Newborn with a gestational age between 23+0 and 29+6 weeks
- Informed consent form signed by the parents

This Document is Confidential.

2.5.2 Exclusion criteria

Patients were excluded from participation into the trial if they met one or more of the following main criteria:

- Major congenital malformations
- Foetal hydrops
- Pregnancy complicated by placental abruption
- Pregnancy complicated by Rh isoimmunization
- Twin pregnancy
- Short umbilical cord*

*This criterion was removed in the amended version 4.0 of the protocol, dated December 22, 2016, due to the recurring inability, discovered in the first part of the trial, to identify cases with a short umbilical cord before randomization.

2.6 INTERVENTIONAL GROUPS

Preterm infants were randomly allocated to one of the two interventional arms reported in Table 1.

Table 1: The two interventional arms of the study.

Arm	Intervention
**A**	**Control:** assistance on the neonatal resuscitation table, after performing cord milking followed by umbilical cord clamping
**B**	**Experimental:** assistance with intact placental circulation, keeping the newborn near the mother's bed at the same level as the placenta

2.7 SAMPLE SIZE

Assuming a proportion of events (grade 3 or 4 IVH, moderate or severe BPD or death from any cause) equal to 47% in the control arm and equal or less than 28% in the experimental arm, corresponding to a relative reduction of the unfavourable events equal to 40%, it was calculated that a sample size of 200 preterm infants (100 per group) would have guaranteed a study power equal to 80%, with a two-sided alpha error equal to 5%, for a standard chi-square test for heterogeneity.

2.8 TREATMENT ALLOCATION

Assignment of each preterm infant to one of the two interventional groups has been performed using a minimization algorithm, with an allocation ratio equal to 1:1.

Minimization is a dynamic randomization algorithm designed to minimize imbalance between treatments taking stratification factors into account. Based on characteristics of

This Document is Confidential.

the current patient and treatment assignments and characteristics of enrolled patients an imbalance score is computed. In the present study, the assignment was performed using a randomization list with an allocation ratio equal to 1:1 in absence of imbalance. A random component in intervention selection was also used in case of imbalance. Indeed, in this situation, a randomization list with an allocation ratio equal to 1:5 in favour of the intervention with the highest imbalance score was used.

The gestational age (23+0 to 26+6 versus 27+0 to 29+6) has been used as a stratification factor.

Treatment allocation process has been carried out at all participating centres by a restricted access web-based system (https://www.eclintrials.org/ect), administered by the Clinical Epidemiology Unit of the IRCCS Ospedale Policlinico San Martino, Genoa, Italy, active 24-hour, and 365 days a year.

2.9 BLINDING AND UNBLINDING

No procedures for treatment masking have been used in the study, however the present SAP has been written by a statistician blinded to treatment allocations and study results.

3 POPULATIONS OF ANALYSIS

The populations of analysis were not originally identified in the study protocol.

The primary and secondary efficacy analyses will be performed on the intention-to-treat (ITT) population. The same evaluations will be repeated on the per-protocol population, as sensitivity analyses.

The safety analyses will be performed on the safety population.

3.1 RANDOMIZED POPULATION

The randomized population will include all randomized preterm infants. Patients will be reported under their randomized intervention arm.

3.2 INTENTION-TO-TREAT POPULATION

The intention-to-treat population will include all randomized preterm infants. Patients erroneously registered twice or more into the study will be accounted for only once. Patients will be analysed according to the treatment assigned at the end of the randomization procedure. For patients registered twice or more the first assigned treatment should be taken as reference.

This Document is Confidential.

3.3 PER-PROTOCOL POPULATION

The per-protocol population will include all randomized preterm infants with no violation of eligibility criteria and interventional protocol. Preterm infants non-assisted with the procedure assigned by the minimization algorithm will be excluded from the per-protocol population.

3.4 SAFETY POPULATION

The safety population will include all randomized preterm infants who have received assistance according to one of the two interventional protocol procedures. Patients will be analysed according to type of assistance they received.

3.5 TREATMENT MISALLOCATIONS

If a preterm infant was:

- randomized but was assisted with the alternative study procedure, then he/she will be anyway reported under his/her randomized interventional group for primary and secondary efficacy analyses but will be reported under the treatment he/she received for all safety analyses.
- randomized but was assisted with an off-protocol procedure, then he/she will be reported under his randomized interventional group for all primary and secondary efficacy analyses and will be excluded from the safety analyses.
- preterm infants in which a treatment misallocation occurred, by definition, will not be included in the per-protocol analyses.

3.6 PROTOCOL DEVIATIONS

The full list of protocol deviations to be included in the study report, those assessed either prior to randomization or after randomization, will be compiled prior to database closure, following the blinded evaluation of the study coordinator.

4 ENDPOINTS AND COVARIATES

4.1 EFFICACY VARIABLES

4.1.1 Primary efficacy variable

The primary efficacy variable is a composite endpoint that consists of grade 3-4 IVH, moderate/severe BPD, and death at 36 weeks of post-conceptional age.

IVH and BPD were diagnosed following the criteria of Papile (19) and Jobe and Bancalari (20), respectively.

This Document is Confidential.

4.1.2 Secondary efficacy variables

The secondary efficacy variables are:

- The single components of the composite endpoint
- The variables reported in list below:

    1 min Apgar score
    5 mins Apgar score
    FiO2 max in delivery room
    Use of neopuff
    Use of physiological solution
    Use of sodium bicarbonate
    Body temperature (°C) at intensive care admission
    Surgical treatment for patent ductus arteriosus (PDA)
    Incidence of IVH
    Use of non-invasive mechanical ventilation
    Duration of non-invasive mechanical ventilation (days)
    Use of invasive mechanical ventilation
    Duration of invasive mechanical ventilation (days)
    iNO administration
    Duration of iNO administration (days)
    Need of erythrocyte concentrate transfusions
    Number of erythrocytes concentrate transfusions
    Use of oxygen therapy
    Duration of oxygen therapy (days)
    FiO2 max during oxygen therapy (%)
    Dopamine use in the first 24 hours
    Dobutamine use in the first 24 hours
    Overall duration of hospitalization (days)
    3-hour cerebral oxygenation
    6-hour cerebral oxygenation
    12-hour cerebral oxygenation
    18-hour cerebral oxygenation
    24-hour cerebral oxygenation
    Superior vena cava blood flow in the first 24 hours (ml/Kg/min)
    PAP value (mmHg)
    Right ventricular ejection (ml/Kg/min)
    Left ventricular ejection (ml/Kg/min)
    Presence of transductal shunt
    Hb max (g/dl) in the first 24 hours
    Ht max (%) in the first 24 hours
    3-hour average PAS (mmHg)
    6-hour average PAS (mmHg)
    12-hour average PAS (mmHg)
    18-hour average PAS (mmHg)
    24-hour average PAS (mmHg)

This Document is Confidential.

S/D AMS
PI AMS
RI AMS
AMS diastolic flow
S/D ACA
PI ACA
RI ACA
ACA diastolic flow

4.2 SAFETY VARIABLES

Safety of the study interventional procedures will be evaluated by the following variables:

Intubation in delivery room
Compressions in delivery room
Use of i.v. adrenaline in delivery room
Use of ET adrenaline in delivery room
Use of surfactant in delivery room
Total number of surfactant doses
CRIB II score
Max serum bilirubin
Exchange transfusions
Need of mechanical ventilation in the first 24 hours
Duration of mechanical ventilation in the first 24 hours (hrs)
Pharmacological closure of PDA
Incidence of NEC
Incidence of PLV
Incidence of IVH
Incidence of ROP
Incidence of sepsis

4.3 HANDLING OF MISSING VALUES

Generally, subjects with missing value for a particular endpoint will contribute to the specific patient population and will be reported in all analyses as a special category ("missing value").

5 STATISTICAL AND ANALYTICAL METHODS

5.1 INTERIM ANALYSES, FINAL ANALYSES AND UNBLINDING

One *interim analysis* has been planned in the study protocol. However, the primary aim of this analysis was to evaluate the feasibility of the experimental intervention on a limited number of preterm infants and did not involve any of the primary and secondary

This Document is Confidential.

efficacy variables. For this reason, no one statistical procedure for the control of the type I error rate was adopted.

The *final analyses* will be performed after the official database release. The database will be locked at the end of all data entry and data cleaning procedures and when the patients' disposition in the study populations will be definitely defined in accordance with the study coordinator.

All statistical analyses will be performed with the statistical package SAS System 9.4.

Unblinding is not applicable to the present study.

5.2 STATISTICAL ANALYSES

Planned data display is reported in Appendix I. Tables referring to the PP population analyses have not been included in the data display since the PP analyses duplicate the ITT analyses on a different study population.

5.2.1 Patients' characteristics at baseline

Description of all baseline characteristics will be presented by interventional group. Data will be reported for the ITT and PP populations.

Discrete variables will be summarized by frequencies and percentages. Percentages will be calculated according to the total number of patients assigned to each interventional group and to the total number of patients included in the specific analysis population.

Continuous variables will be summarized by use of standard measures of central tendency and dispersion: mean, standard deviation (SD), standard error (SE), median, minimum, maximum, interquartile range (IQR).

No formal test will be performed to compare patients' characteristics at baseline.

5.2.2 Primary efficacy analysis

Data regarding the primary efficacy variable will be presented by treatment group and will be reported for the ITT and PP populations. Evaluations on the PP population would be considered as sensitivity analyses.

The distribution of patients by the incidence of composite endpoint will be summarized by frequencies and percentages. Percentages will be calculated according to the total number of patients assigned to each interventional group and to the total number of patients included in the specific analysis population. The 95% CI of the percentage will be calculated according to the exact method.

The proportions of events occurred in the two interventional arms will be compared with the chi-square test for heterogeneity or the Fisher's exact test, if appropriate.

The estimate of the odds ratio and its 95% Wald CI will be obtained with a logistic regression model with the interventional arm as independent covariate. The umbilical

This Document is Confidential.

cord milking arm will be treated as reference group. The Wald chi-square test will be used to test the effect of the experimental intervention.

With the intent to verify if a heterogeneity of the experimental treatment effect exists in strata identify by gestational age (23-26 wks. versus 27-29 wks.) and type of delivery (vaginal versus caesarean), two subgroup analyses will be performed with a logistic regression model including the interventional group, the stratification variable, and their interaction term. The odds ratios of event and their 95% Wald CI will be estimated in each stratum and the interaction test will be used to test the heterogeneity of the strata-specific treatment effects.

No adjustment for multiplicity will be adopted.

5.2.3 Secondary efficacy analyses

Data regarding the secondary efficacy variables will be presented by treatment group and will be reported for the ITT and PP populations. Evaluations on the PP population would be considered as sensitivity analyses.

The statistical analysis of the single components of the composite endpoint will be conducted with the same approach adopted for the primary efficacy variable.

The other secondary efficacy variables will be analysed according to their type.

Discrete variables will be summarized by frequencies and percentages. Percentages will be calculated according to the total number of patients assigned to each interventional group and to the total number of patients included in the specific analysis population. The proportions of events occurred in the two interventional arms will be compared with the chi-square test for heterogeneity or the Fisher’s exact test, if appropriate.

Continuous variables will be summarized by use of standard measures of central tendency and dispersion: mean, standard deviation (SD), standard error (SE), median, minimum, maximum, interquartile range (IQR). The average values of each parameter observed in the two interventional arms will be compared by means of the Wilcoxon two-sample test.

No adjustment for multiplicity will be adopted.

5.2.4 Safety Analyses

Data regarding the safety variables will be presented by treatment group and will be reported for the safety and PP populations. Evaluations on the PP population would be considered as sensitivity analyses.

The statistical analysis of the safety parameters will be conducted with the same approach adopted for the other secondary efficacy variable.

6 REFERENCES

[1] Rabe H et al. A Systematic Review and Meta-Analysis of a Brief Delay in Clamping the Umbilical Cord of Preterm Infants. Neonatology 2008; 93:138–144

This Document is Confidential.

[2] Mercer JS et al. Delayed Cord Clamping in Very Preterm Infants Reduces the Incidence of Intraventricular Hemorrhage and Late-Onset Sepsis: A Randomized,Controlled Trial. Pediatrics 2006;117;1235-1242

[3] Wyllie J, Niermeyer S. The role of resuscitation drugs and placental transfusion in the delivery room management of newborn infants. Seminars in fetal and neonatal medicine 2008, 1-8

[4] Van Rheenen PF, Brabin BJ. A practical approach to timing cord clamping in resource poor settings. BMJ 2006; 333:954-958

[5] Aladangady N, McHugh S, Aitchison TC, et al. Infants' blood volume in a controlled trial of placental transfusion at preterm delivery. Pediatrics. 2006;117(1):93-8

[6] Bhatt S, Alison BJ, Wallace EM, et al. Delaying cord clamping until ventilation onset improves cardiovascular function at birth in preterm lambs. J Physiol. 2013:15;591(Pt 8):2113-26

[7] Meyer MP, Mildenhall L. Delayed cord clamping and blood flow in the superior vena cava in preterm infants: an observational study. Arch Dis Child Fetal Neonatal Ed. 2012; 97(6):F484-6

[8] Vento M, Cheung PY, Aguar M. The first golden minutes of the extremely-low-gestationalage neonate: a gentle approach. Neonatology. 2009;95(4):286-98

[9] Kattwinkel J, Perlman JM, Aziz K, et al. Neonatal resuscitation: 2010 American Heart Association Guidelines for Cardiopulmonary Resuscitation and Emergency Cardiovascular Care. Pediatrics. 2010;126(5):e1400-13.

[10] Rabe H, Jewison A, Alvarez RF, Crook D, Stilton D, Bradley R, Holden D; Brighton Perinatal Study Group. Milking compared with delayed cord clamping to increase placental transfusion in preterm neonates: a randomized controlled trial. Obstet Gynecol. 2011;117(2 Pt 1):205-11

[11] March MI, Hacker MR, Parson AW et al. The effects of umbilical cord milking in extremely preterm infants: a randomized controlled trial. J Perinatol. 2013;33(10):763-7

[12] Takami T, Suganami Y, Sunohara D, et al. Umbilical cord milking stabilizes cerebral oxygenation and perfusion in infants born before 29 weeks of gestation. J Pediatr. 2012;161(4):742-7 19

[13] Hosono S, Mugishima H, Fujita H, et al. Blood pressure and urine output during the first 120 h of life in infants born at less than 29 weeks' gestation related to umbilical cord milking. Arch Dis Child Fetal Neonatal Ed. 2009;94(5):F328-31

[14] Oh W et al. Effects of delayed cord clamping in very-low-birth-weight infants. J of Perinatology 2011; 31: S68–S71

[15] Mercer JS et al. Seven-month developmental outcomes of very low birth weight infants enrolled in a randomized controlled trial of delayed versus immediate cord clamping. Journal of Perinatology 2010; 30: 11–16

This Document is Confidential.

[16] van Rheenen P. Delayed cord clamping and improved infant outcomes. BMJ 2011: 15; 343:d7127

[17] Niermeyer S, Velaphi S. Promoting physiologic transition at birth: Re-examining resuscitation and the timing of cord clamping. Semin Fetal Neonatal Med. 2013;18(6):385- 92.

[18] Pushpa-Rajah A Bradshaw L, Dorling J, et al. Cord pilot trial - immediate versus deferred cord clamping for very preterm birth (before 32 weeks gestation): study protocol for a randomized controlled trial. Trials 2014, 15:258

[19] Papile LS, Burstein J, Burstein R, Koffler H. Incidence and evolution of the subependymal intraventricular hemorrhage: a study of infants weighing less than 1500 grams. J Pediatr. 1978, 92:529–34

[20] Jobe A, Bancalari E: Bronchopulmonary dysplasia. Am J Respir Crit Care Med 2001, 163:1723–1729.

This Document is Confidential.

APPENDIX 1 - PLANNED DATA DISPLAY

This Document is Confidential.

NOTE

(1) Chi-square tests, measures based on chi-square and Fisher's exact test are reported below the primary table.

(2) The proportions of events observed in each arm, their 95% exact confidence intervals, the difference between the two proportions and its exact confidence interval are reported below the primary table.

(3) The standard output of the logistic regression model is reported below the primary table.

(4) The standard output of the logistic regression model including the interaction term between interventional arm and stratification factor is reported below the primary table.

(5) The standard output of the Wilcoxon rank-sum test is reported below the primary table.

This Document is Confidential.

1. STUDY POPULATION

TABLE 1.1: STUDY PERIOD

RANDOMIZED POPULATION

Date of first randomization	Date of last randomization	Date of last discharge

This Document is Confidential.

TABLE 1.2: PATIENTS DISPOSITION

Population	Arm A (N)	Arm B (N)	Total (N)	Total (%)
Randomized				
Intention-to-treat				
Safety				
Per-protocol				

NOTE: Arm A = UMBELICAL CORD MILKING, Arm B = DELAYED UMBELICAL CORD CLAMPING

This Document is Confidential.

TABLE 1.3: LISTING OF PATIENTS NOT INCLUDED IN THE INTENTION-TO-TREAT POPULATION

Patient no.	Centre	Randomization date	Treatment arm	Reason

NOTE: Arm A = UMBELICAL CORD MILKING, Arm B = DELAYED UMBELICAL CORD CLAMPING

This Document is Confidential.

TABLE 1.4: DISTRIBUTION OF PATIENTS BY CENTRE AND TREATMENT ARM

INTENTION-TO-TREAT POPULATION

	Treatment Arm				Total	
	Arm A		Arm B			
	N	%	N	%	N	%
Centre						
BARI						
BOLOGNA						
FIRENZE						
MILANO						
MONZA						
PERUGIA						
RIMINI						
VICENZA						
Total						

NOTE: Arm A = UMBELICAL CORD MILKING, Arm B = DELAYED UMBELICAL CORD CLAMPING

This Document is Confidential.

TABLE 1.5: LISTING OF PATIENTS NOT INCLUDED IN THE SAFETY POPULATION

Patient no.	Centre	Randomization date	Treatment arm	Reason

NOTE: Arm A = UMBELICAL CORD MILKING, Arm B = DELAYED UMBELICAL CORD CLAMPING

This Document is Confidential.

TABLE 1.6: DISTRIBUTION OF PATIENTS BY TREATMENT ACTUALLY RECEIVED AND TREATMENT ARM

INTENTION-TO-TREAT POPULATION

	Treatment Arm				Total	
	Arm A		Arm B			
	N	%	N	%	N	%
Treatment actually received						
Arm A						
Arm B						
Other						
Total						

NOTE: Arm A = UMBELICAL CORD MILKING, Arm B = DELAYED UMBELICAL CORD CLAMPING

This Document is Confidential.

TABLE 1.7: LISTING OF PATIENTS NOT TREATED ACCORDING TO THE RANDOMIZATION ASSIGNMENT

Patient no.	Centre	Randomization date	Treatment assigned	Treatment actually received

NOTE: Arm A = UMBELICAL CORD MILKING, Arm B = DELAYED UMBELICAL CORD CLAMPING

This Document is Confidential.

TABLE 1.8: LISTING OF PATIENTS NOT INCLUDED IN THE PER-PROTOCOL POPULATION

Patient no.	Centre	Randomization date	Treatment arm	Reason

NOTE: Arm A = UMBELICAL CORD MILKING, Arm B = DELAYED UMBELICAL CORD CLAMPING

This Document is Confidential.

2. PATIENTS CHARACTERISTICS AT BASELINE

TABLE 2.1: DISTRIBUTION OF PATIENTS BY SEX AND TREATMENT ARM

INTENTION-TO-TREAT POPULATION

	Treatment Arm				Total	
	Arm A		Arm B			
	N	%	N	%	N	%
Sex						
Male						
Female						
Missing value						
Total						

NOTE: Arm A = UMBELICAL CORD MILKING, Arm B = DELAYED UMBELICAL CORD CLAMPING

This Document is Confidential.

TABLE 2.2: GESTATIONAL AGE BY TREATMENT ARM

INTENTION-TO-TREAT POPULATION

		Treatment Arm		Total
		Arm A	Arm B	
Gestational age (wks)	N			
	Missing			
	Mean			
	SD			
	Median			
	Minimum			
	First quartile			
	Third quartile			
	Maximum			

NOTE: Arm A = UMBELICAL CORD MILKING, Arm B = DELAYED UMBELICAL CORD CLAMPING

This Document is Confidential.

TABLE 2.3: DISTRIBUTION OF PATIENTS BY GESTATIONAL AGE AND TREATMENT ARM

INTENTION-TO-TREAT POPULATION

	Treatment Arm				Total	
	Arm A		Arm B			
	N	%	N	%	N	%
Gestational age						
23-26 wks						
27-29 wks						
Missing value						
Total						

NOTE: Arm A = UMBELICAL CORD MILKING, Arm B = DELAYED UMBELICAL CORD CLAMPING

This Document is Confidential.

TABLE 2.4: BIRTHWEIGHT BY TREATMENT ARM

INTENTION-TO-TREAT POPULATION

		Treatment Arm		
		Arm A	Arm B	Total
Weight (g)	N			
	Missing			
	Mean			
	SD			
	Median			
	Minimum			
	First quartile			
	Third quartile			
	Maximum			

NOTE: Arm A = UMBELICAL CORD MILKING, Arm B = DELAYED UMBELICAL CORD CLAMPING

This Document is Confidential.

TABLE 2.5: DISTRIBUTION OF PATIENTS BY TYPE OF DELIVERY AND TREATMENT ARM

INTENTION-TO-TREAT POPULATION

	Treatment Arm				Total	
	Arm A		Arm B			
	N	%	N	%	N	%
Type of delivery						
Natural vaginal delivery						
Induced vaginal delivery						
Caesarean delivery						
Missing value						
Total						

NOTE: Arm A = UMBELICAL CORD MILKING, Arm B = DELAYED UMBELICAL CORD CLAMPING

This Document is Confidential.

TABLE 2.6: DISTRIBUTION OF PATIENTS BY USE OF OXYTOCIN AND TREATMENT ARM

INTENTION-TO-TREAT POPULATION

	Treatment Arm				Total	
	Arm A		Arm B			
	N	%	N	%	N	%
Use of oxytocin						
No						
Yes, i.v.						
Yes, i.m.						
Missing value						
Total						

NOTE: Arm A = UMBELICAL CORD MILKING, Arm B = DELAYED UMBELICAL CORD CLAMPING

This Document is Confidential.

TABLE 2.7: DISTRIBUTION OF PATIENTS BY TIMING OF OXYTOCIN ADMINISTRATION AND TREATMENT ARM

INTENTION-TO-TREAT POPULATION

	Treatment Arm				Total	
	Arm A		Arm B			
	N	%	N	%	N	%
Timing of oxytocin administration						
No oxytocin						
Before cord clamping						
After cord clamping						
Before and after cord clamping						
Missing value						
Total						

NOTE: Arm A = UMBELICAL CORD MILKING, Arm B = DELAYED UMBELICAL CORD CLAMPING

This Document is Confidential.

TABLE 2.8: DISTRIBUTION OF PATIENTS BY USE OF ANTENAL STEROIDS AND TREATMENT ARM

INTENTION-TO-TREAT POPULATION

	Treatment Arm				Total	
	Arm A		Arm B			
	N	%	N	%	N	%
Use of steroids						
No						
Yes						
Missing value						
Total						

NOTE: Arm A = UMBELICAL CORD MILKING, Arm B = DELAYED UMBELICAL CORD CLAMPING

This Document is Confidential.

TABLE 2.9: DISTRIBUTION OF PATIENTS BY PRESENCE OF MATERNAL COMPLICATIONS DURING PREGNANCY AND TREATMENT ARM

INTENTION-TO-TREAT POPULATION

	Treatment Arm				Total	
	Arm A		Arm B			
	N	%	N	%	N	%
Maternal complications						
No						
Yes						
Missing value						
Total						

NOTE: Arm A = UMBELICAL CORD MILKING, Arm B = DELAYED UMBELICAL CORD CLAMPING

This Document is Confidential.

TABLE 2.10: DISTRIBUTION OF PATIENTS BY PROM AND TREATMENT ARM

INTENTION-TO-TREAT POPULATION

	Treatment Arm				Total	
	Arm A		Arm B			
	N	%	N	%	N	%
PROM						
No						
Yes						
Missing value						
Total						

NOTE: Arm A = UMBELICAL CORD MILKING, Arm B = DELAYED UMBELICAL CORD CLAMPING

This Document is Confidential.

TABLE 2.11: DISTRIBUTION OF PATIENTS BY CLINICAL CHORIOAMNIONITIS AND TREATMENT ARM

INTENTION-TO-TREAT POPULATION

	Treatment Arm				Total	
	Arm A		Arm B			
	N	%	N	%	N	%
Chorioamnionitis						
No						
Yes						
Missing value						
Total						

NOTE: Arm A = UMBELICAL CORD MILKING, Arm B = DELAYED UMBELICAL CORD CLAMPING

This Document is Confidential.

TABLE 2.12: DISTRIBUTION OF PATIENTS BY MATERNAL PREECLAMPSIA AND TREATMENT ARM

INTENTION-TO-TREAT POPULATION

	Treatment Arm				Total	
	Arm A		Arm B			
	N	%	N	%	N	%
Preeclampsia						
No						
Yes						
Missing value						
Total						

NOTE: Arm A = UMBELICAL CORD MILKING, Arm B = DELAYED UMBELICAL CORD CLAMPING

This Document is Confidential.

TABLE 2.13: DISTRIBUTION OF PATIENTS BY IDIOPATHIC PRETERM DELIVERY AND TREATMENT ARM

INTENTION-TO-TREAT POPULATION

	Treatment Arm				Total	
	Arm A		Arm B			
	N	%	N	%	N	%
Idiopathic preterm delivery						
No						
Yes						
Missing value						
Total						

NOTE: Arm A = UMBELICAL CORD MILKING, Arm B = DELAYED UMBELICAL CORD CLAMPING

This Document is Confidential.

TABLE 2.14: DISTRIBUTION OF PATIENTS BY IUGR AND TREATMENT ARM

INTENTION-TO-TREAT POPULATION

	Treatment Arm				Total	
	Arm A		Arm B			
	N	%	N	%	N	%
IUGR						
No						
Yes						
Missing value						
Total						

NOTE: Arm A = UMBELICAL CORD MILKING, Arm B = DELAYED UMBELICAL CORD CLAMPING

This Document is Confidential.

TABLE 2.15: DISTRIBUTION OF PATIENTS BY MATERNAL ANESTHESIA AND TREATMENT ARM

INTENTION-TO-TREAT POPULATION

	Treatment Arm				Total	
	Arm A		Arm B			
	N	%	N	%	N	%
Maternal anaesthesia						
No						
Yes, epidural						
Yes, other						
Missing value						
Total						

NOTE: Arm A = UMBELICAL CORD MILKING, Arm B = DELAYED UMBELICAL CORD CLAMPING

This Document is Confidential.

TABLE 2.16: CORD CLAMPING TIME BY TREATMENT ARM

INTENTION-TO-TREAT POPULATION

		Treatment Arm		Total
		Arm A	Arm B	
Time (secs)	N			
	Missing			
	Mean			
	SD			
	Median			
	Minimum			
	First quartile			
	Third quartile			
	Maximum			

NOTE: Arm A = UMBELICAL CORD MILKING, Arm B = DELAYED UMBELICAL CORD CLAMPING

This Document is Confidential.

3. PRIMARY COMPOSITE OUTCOME ANALYSES

TABLE 3.1: DISTRIBUTION OF PATIENTS BY INCIDENCE OF UNFAVOURABLE OUTCOME AND TREATMENT ARM

INTENTION-TO-TREAT POPULATION

	Treatment Arm				Total	
	Arm A		Arm B			
	N	%	N	%	N	%
Unfavourable outcome #						
No events						
IVH						
BPD						
IVH + BPD						
Death						
IVH + death						
BPD + death						
IVH + BPD + death						
Missing value						
Total						

Accounts for grade 3 or 4 IVH, moderate or severe BPD, or death

NOTE: Arm A = UMBELICAL CORD MILKING, Arm B = DELAYED UMBELICAL CORD CLAMPING

This Document is Confidential.

TABLE 3.2: DISTRIBUTION OF PATIENTS BY INCIDENCE OF UNFAVOURABLE OUTCOME TREATMENT ARM (1, 2, 3)

INTENTION-TO-TREAT POPULATION

	Treatment Arm				Total	
	Arm A		Arm B			
	N	%	N	%	N	%
Unfavourable outcome #						
No						
Yes						
Missing value						
Total						

Accounts for grade 3 or 4 IVH, moderate or severe BPD, or death

NOTE: Arm A = UMBELICAL CORD MILKING, Arm B = DELAYED UMBELICAL CORD CLAMPING

This Document is Confidential.

TABLE 3.3: DISTRIBUTION OF PATIENTS BY INCIDENCE OF UNFAVOURABLE OUTCOME TREATMENT ARM (2)

INTENTION-TO-TREAT POPULATION

GESTATIONAL AGE 23-26 WEEKS AT BIRTH

	Treatment Arm				Total	
	Arm A		Arm B			
	N	%	N	%	N	%
Unfavourable outcome #						
No						
Yes						
Missing value						
Total						

Accounts for grade 3 or 4 IVH, moderate or severe BPD, or death

NOTE: Arm A = UMBELICAL CORD MILKING, Arm B = DELAYED UMBELICAL CORD CLAMPING

This Document is Confidential.

TABLE 3.4: DISTRIBUTION OF PATIENTS BY INCIDENCE OF UNFAVOURABLE OUTCOME TREATMENT ARM (2, 4)

INTENTION-TO-TREAT POPULATION

GESTATIONAL AGE 27-29 WEEKS AT BIRTH

	Treatment Arm				Total	
	Arm A		Arm B			
	N	%	N	%	N	%
Unfavourable outcome #						
No						
Yes						
Missing value						
Total						

Accounts for grade 3 or 4 IVH, moderate or severe BPD, or death

NOTE: Arm A = UMBELICAL CORD MILKING, Arm B = DELAYED UMBELICAL CORD CLAMPING

This Document is Confidential.

TABLE 3.5: DISTRIBUTION OF PATIENTS BY INCIDENCE OF UNFAVOURABLE OUTCOME TREATMENT ARM (2)

INTENTION-TO-TREAT POPULATION

VAGINAL DELIVERY

	Treatment Arm				Total	
	Arm A		Arm B			
	N	%	N	%	N	%
Unfavourable outcome #						
No						
Yes						
Missing value						
Total						

Accounts for grade 3 or 4 IVH, moderate or severe BPD, or death

NOTE: Arm A = UMBELICAL CORD MILKING, Arm B = DELAYED UMBELICAL CORD CLAMPING

This Document is Confidential.

TABLE 3.6: DISTRIBUTION OF PATIENTS BY INCIDENCE OF UNFAVOURABLE OUTCOME TREATMENT ARM (2, 4)

INTENTION-TO-TREAT POPULATION

CESAREAN DELIVERY

	Treatment Arm				Total	
	Arm A		Arm B			
	N	%	N	%	N	%
Unfavourable outcome #						
No						
Yes						
Missing value						
Total						

Accounts for grade 3 or 4 IVH, moderate or severe BPD, or death

NOTE: Arm A = UMBELICAL CORD MILKING, Arm B = DELAYED UMBELICAL CORD CLAMPING

This Document is Confidential.

4. PRIMARY OUTCOMES ANALYSES

TABLE 4.1: DISTRIBUTION OF PATIENTS BY INCIDENCE OF GRADE 3 OR 4 IVH AND TREATMENT ARM (1, 2, 3)

INTENTION-TO-TREAT POPULATION

	Treatment Arm				Total	
	Arm A		Arm B			
	N	%	N	%	N	%
IVH						
No						
Yes						
Missing value						
Total						

NOTE: Arm A = UMBELICAL CORD MILKING, Arm B = DELAYED UMBELICAL CORD CLAMPING

This Document is Confidential.

TABLE 4.2: DISTRIBUTION OF PATIENTS BY INCIDENCE OF GRADE 3 OR 4 IVH AND TREATMENT ARM (2)

INTENTION-TO-TREAT POPULATION

GESTATIONAL AGE 23-26 WEEKS AT BIRTH

	Treatment Arm				Total	
	Arm A		Arm B			
	N	%	N	%	N	%
IVH						
No						
Yes						
Missing value						
Total						

NOTE: Arm A = UMBELICAL CORD MILKING, Arm B = DELAYED UMBELICAL CORD CLAMPING

This Document is Confidential.

TABLE 4.3: DISTRIBUTION OF PATIENTS BY INCIDENCE OF GRADE 3 OR 4 IVH AND TREATMENT ARM (2, 4)

INTENTION-TO-TREAT POPULATION

GESTATIONAL AGE 27-29 WEEKS AT BIRTH

	Treatment Arm				Total	
	Arm A		Arm B			
	N	%	N	%	N	%
IVH						
No						
Yes						
Missing value						
Total						

NOTE: Arm A = UMBELICAL CORD MILKING, Arm B = DELAYED UMBELICAL CORD CLAMPING

This Document is Confidential.

TABLE 4.4: DISTRIBUTION OF PATIENTS BY INCIDENCE OF GRADE 3 OR 4 IVH AND TREATMENT ARM (2)

INTENTION-TO-TREAT POPULATION

VAGINAL DELIVERY

	Treatment Arm				Total	
	Arm A		Arm B			
	N	%	N	%	N	%
IVH						
No						
Yes						
Missing value						
Total						

NOTE: Arm A = UMBELICAL CORD MILKING, Arm B = DELAYED UMBELICAL CORD CLAMPING

This Document is Confidential.

TABLE 4.5: DISTRIBUTION OF PATIENTS BY INCIDENCE OF GRADE 3 OR 4 IVH AND TREATMENT ARM (2, 4)

INTENTION-TO-TREAT POPULATION

CESAREAN DELIVERY

	Treatment Arm				Total	
	Arm A		Arm B			
	N	%	N	%	N	%
IVH						
No						
Yes						
Missing value						
Total						

NOTE: Arm A = UMBELICAL CORD MILKING, Arm B = DELAYED UMBELICAL CORD CLAMPING

This Document is Confidential.

TABLE 4.6: DISTRIBUTION OF PATIENTS BY INCIDENCE OF MODERATE OR SEVERE BPD AND TREATMENT ARM (1, 2, 3)

INTENTION-TO-TREAT POPULATION

	Treatment Arm					
	Arm A		Arm B		Total	
	N	%	N	%	N	%
BPD						
No						
Yes						
Missing value						
Total						

NOTE: Arm A = UMBELICAL CORD MILKING, Arm B = DELAYED UMBELICAL CORD CLAMPING

This Document is Confidential.

TABLE 4.7: DISTRIBUTION OF PATIENTS BY INCIDENCE OF MODERATE OR SEVERE BPD AND TREATMENT ARM (2)

INTENTION-TO-TREAT POPULATION

GESTATIONAL AGE 23-26 WEEKS AT BIRTH

	Treatment Arm				Total	
	Arm A		Arm B			
	N	%	N	%	N	%
BPD						
No						
Yes						
Missing value						
Total						

NOTE: Arm A = UMBELICAL CORD MILKING, Arm B = DELAYED UMBELICAL CORD CLAMPING

This Document is Confidential.

TABLE 4.8: DISTRIBUTION OF PATIENTS BY INCIDENCE OF MODERATE OR SEVERE BPD AND TREATMENT ARM (2, 4)

INTENTION-TO-TREAT POPULATION

GESTATIONAL AGE 27-29 WEEKS AT BIRTH

	Treatment Arm				Total	
	Arm A		Arm B			
	N	%	N	%	N	%
BPD						
No						
Yes						
Missing value						
Total						

NOTE: Arm A = UMBELICAL CORD MILKING, Arm B = DELAYED UMBELICAL CORD CLAMPING

This Document is Confidential.

TABLE 4.9: DISTRIBUTION OF PATIENTS BY INCIDENCE OF MODERATE OR SEVERE BPD AND TREATMENT ARM (2)

INTENTION-TO-TREAT POPULATION

VAGINAL DELIVERY

	Treatment Arm				Total	
	Arm A		Arm B			
	N	%	N	%	N	%
BPD						
No						
Yes						
Missing value						
Total						

NOTE: Arm A = UMBELICAL CORD MILKING, Arm B = DELAYED UMBELICAL CORD CLAMPING

This Document is Confidential.

TABLE 4.10: DISTRIBUTION OF PATIENTS BY INCIDENCE OF MODERATE OR SEVERE BPD AND TREATMENT ARM (2, 4)

INTENTION-TO-TREAT POPULATION

CESAREAN DELIVERY

	Treatment Arm				Total	
	Arm A		Arm B			
	N	%	N	%	N	%
BPD						
No						
Yes						
Missing value						
Total						

NOTE: Arm A = UMBELICAL CORD MILKING, Arm B = DELAYED UMBELICAL CORD CLAMPING

This Document is Confidential.

TABLE 4.11: DISTRIBUTION OF PATIENTS BY INCIDENCE OF DEATH AND TREATMENT ARM (1, 2, 3)

INTENTION-TO-TREAT POPULATION

	Treatment Arm				Total	
	Arm A		Arm B			
	N	%	N	%	N	%
Death						
No						
Yes						
Missing value						
Total						

NOTE: Arm A = UMBELICAL CORD MILKING, Arm B = DELAYED UMBELICAL CORD CLAMPING

This Document is Confidential.

TABLE 4.12: DISTRIBUTION OF PATIENTS BY INCIDENCE OF DEATH AND TREATMENT ARM (2)

INTENTION-TO-TREAT POPULATION

GESTATIONAL AGE 23-26 WEEKS AT BIRTH

	Treatment Arm				Total	
	Arm A		Arm B			
	N	%	N	%	N	%
Death						
No						
Yes						
Missing value						
Total						

NOTE: Arm A = UMBELICAL CORD MILKING, Arm B = DELAYED UMBELICAL CORD CLAMPING

This Document is Confidential.

TABLE 4.13: DISTRIBUTION OF PATIENTS BY INCIDENCE OF DEATH AND TREATMENT ARM (2, 4)

INTENTION-TO-TREAT POPULATION

GESTATIONAL AGE 27-29 WEEKS AT BIRTH

	Treatment Arm				Total	
	Arm A		Arm B			
	N	%	N	%	N	%
Death						
No						
Yes						
Missing value						
Total						

NOTE: Arm A = UMBELICAL CORD MILKING, Arm B = DELAYED UMBELICAL CORD CLAMPING

This Document is Confidential.

TABLE 4.14: DISTRIBUTION OF PATIENTS BY INCIDENCE OF DEATH AND TREATMENT ARM (2)

INTENTION-TO-TREAT POPULATION

VAGINAL DELIVERY

	Treatment Arm				Total	
	Arm A		Arm B			
	N	%	N	%	N	%
Death						
No						
Yes						
Missing value						
Total						

NOTE: Arm A = UMBELICAL CORD MILKING, Arm B = DELAYED UMBELICAL CORD CLAMPING

This Document is Confidential.

TABLE 4.15: DISTRIBUTION OF PATIENTS BY INCIDENCE OF DEATH AND TREATMENT ARM (2, 4)

INTENTION-TO-TREAT POPULATION

CESAREAN DELIVERY

	Treatment Arm				Total	
	Arm A		Arm B			
	N	%	N	%	N	%
Death						
No						
Yes						
Missing value						
Total						

NOTE: Arm A = UMBELICAL CORD MILKING, Arm B = DELAYED UMBELICAL CORD CLAMPING

This Document is Confidential.

5. SECONDARY OUTCOMES ANALYSES

TABLE 5.1: ONE MINUTE APGAR SCORE BY TREATMENT ARM (5)

INTENTION-TO-TREAT POPULATION

		Treatment Arm		Total
		Arm A	Arm B	
1 min Apgar score	N			
	Missing			
	Mean			
	SD			
	Median			
	Minimum			
	First quartile			
	Third quartile			
	Maximum			

NOTE: Arm A = UMBELICAL CORD MILKING, Arm B = DELAYED UMBELICAL CORD CLAMPING

This Document is Confidential.

TABLE 5.2: FIVE MINUTES APGAR SCORE BY TREATMENT ARM (5)

INTENTION-TO-TREAT POPULATION

		Treatment Arm		Total
		Arm A	Arm B	
5 mins Apgar score	N			
	Missing			
	Mean			
	SD			
	Median			
	Minimum			
	First quartile			
	Third quartile			
	Maximum			

NOTE: Arm A = UMBELICAL CORD MILKING, Arm B = DELAYED UMBELICAL CORD CLAMPING

This Document is Confidential.

TABLE 5.3: FiO2 MAX IN DELIVERY ROOM BY TREATMENT ARM (5)

INTENTION-TO-TREAT POPULATION

		Treatment Arm		Total
		Arm A	Arm B	
FiO2 max	N			
	Missing			
	Mean			
	SD			
	Median			
	Minimum			
	First quartile			
	Third quartile			
	Maximum			

NOTE: Arm A = UMBELICAL CORD MILKING, Arm B = DELAYED UMBELICAL CORD CLAMPING

This Document is Confidential.

TABLE 5.4: DISTRIBUTION OF PATIENTS BY USE OF NEOPUFF AND TREATMENT ARM (1)

INTENTION-TO-TREAT POPULATION

	Treatment Arm				Total	
	Arm A		Arm B			
	N	%	N	%	N	%
Use of neopuff						
No						
Yes						
Missing value						
Total						

NOTE: Arm A = UMBELICAL CORD MILKING, Arm B = DELAYED UMBELICAL CORD CLAMPING

This Document is Confidential.

TABLE 5.5: DISTRIBUTION OF PATIENTS BY USE OF PHYSIOLOGICAL SOLUTION AND TREATMENT ARM (1)

INTENTION-TO-TREAT POPULATION

	Treatment Arm				Total	
	Arm A		Arm B			
	N	%	N	%	N	%
Use of physiological solution						
No						
Yes						
Missing value						
Total						

NOTE: Arm A = UMBELICAL CORD MILKING, Arm B = DELAYED UMBELICAL CORD CLAMPING

This Document is Confidential.

TABLE 5.6: DISTRIBUTION OF PATIENTS BY USE OF SODIUM BICARBONATE AND TREATMENT ARM (1)

INTENTION-TO-TREAT POPULATION

	Treatment Arm				Total	
	Arm A		Arm B			
	N	%	N	%	N	%
Use of sodium bicarbonate						
No						
Yes						
Missing value						
Total						

NOTE: Arm A = UMBELICAL CORD MILKING, Arm B = DELAYED UMBELICAL CORD CLAMPING

This Document is Confidential.

TABLE 5.7: BODY TEMPERATURE AT INTENSIVE CARE ADMISSION BY TREATMENT ARM (5)

INTENTION-TO-TREAT POPULATION

		Treatment Arm		Total
		Arm A	Arm B	
Body temperature (C)	N			
	Missing			
	Mean			
	SD			
	Median			
	Minimum			
	First quartile			
	Third quartile			
	Maximum			

NOTE: Arm A = UMBELICAL CORD MILKING, Arm B = DELAYED UMBELICAL CORD CLAMPING

This Document is Confidential.

TABLE 5.8: DISTRIBUTION OF PATIENTS BY INCIDENCE OF RDS AND TREATMENT ARM (1)

INTENTION-TO-TREAT POPULATION

	Treatment Arm				Total	
	Arm A		Arm B			
	N	%	N	%	N	%
RDS						
No						
Yes						
Missing value						
Total						

NOTE: Arm A = UMBELICAL CORD MILKING, Arm B = DELAYED UMBELICAL CORD CLAMPING

This Document is Confidential.

TABLE 5.9: DISTRIBUTION OF PATIENTS BY SURGICAL TREATMENT FOR PDA AND TREATMENT ARM (1)

INTENTION-TO-TREAT POPULATION

	Treatment Arm				Total	
	Arm A		Arm B			
	N	%	N	%	N	%
Surgical treatment						
No						
Yes						
Missing value						
Total						

NOTE: Arm A = UMBELICAL CORD MILKING, Arm B = DELAYED UMBELICAL CORD CLAMPING

This Document is Confidential.

TABLE 5.10: DISTRIBUTION OF PATIENTS BY INCIDENCE OF IVH AND TREATMENT ARM (1)

INTENTION-TO-TREAT POPULATION

	Treatment Arm				Total	
	Arm A		Arm B			
	N	%	N	%	N	%
IVH						
No						
Yes						
Missing value						
Total						

NOTE: Arm A = UMBELICAL CORD MILKING, Arm B = DELAYED UMBELICAL CORD CLAMPING

This Document is Confidential.

TABLE 5.11: DISTRIBUTION OF PATIENTS BY USE OF NON-INVASIVE MECHANICAL VENTILATION AND TREATMENT ARM (1)

INTENTION-TO-TREAT POPULATION

	Treatment Arm				Total	
	Arm A		Arm B			
	N	%	N	%	N	%
Non-invasive mechanical ventilation						
No						
Yes						
Missing value						
Total						

NOTE: Arm A = UMBELICAL CORD MILKING, Arm B = DELAYED UMBELICAL CORD CLAMPING

This Document is Confidential.

TABLE 5.12: DURATION OF NON-INVASIVE MECHANICAL VENTILATION BY TREATMENT ARM (5)

INTENTION-TO-TREAT POPULATION

		Treatment Arm		
		Arm A	Arm B	Total
Duration (days)	N			
	Missing			
	Mean			
	SD			
	Median			
	Minimum			
	First quartile			
	Third quartile			
	Maximum			

NOTE: Arm A = UMBELICAL CORD MILKING, Arm B = DELAYED UMBELICAL CORD CLAMPING

This Document is Confidential.

TABLE 5.13: DISTRIBUTION OF PATIENTS BY USE OF INVASIVE MECHANICAL VENTILATION AND TREATMENT ARM (1)

INTENTION-TO-TREAT POPULATION

	Treatment Arm				Total	
	Arm A		Arm B			
	N	%	N	%	N	%
Invasive mechanical ventilation						
No						
Yes						
Missing value						
Total						

NOTE: Arm A = UMBELICAL CORD MILKING, Arm B = DELAYED UMBELICAL CORD CLAMPING

This Document is Confidential.

TABLE 5.14: DURATION OF INVASIVE MECHANICAL VENTILATION BY TREATMENT ARM (5)

INTENTION-TO-TREAT POPULATION

		Treatment Arm		Total
		Arm A	Arm B	
Duration (days)	N			
	Missing			
	Mean			
	SD			
	Median			
	Minimum			
	First quartile			
	Third quartile			
	Maximum			

NOTE: Arm A = UMBELICAL CORD MILKING, Arm B = DELAYED UMBELICAL CORD CLAMPING

This Document is Confidential.

TABLE 5.15: DISTRIBUTION OF PATIENTS BY iNO ADMINISTRATION AND TREATMENT ARM (1)

INTENTION-TO-TREAT POPULATION

	Treatment Arm				Total	
	Arm A		Arm B			
	N	%	N	%	N	%
iNO						
No						
Yes						
Missing value						
Total						

NOTE: Arm A = UMBELICAL CORD MILKING, Arm B = DELAYED UMBELICAL CORD CLAMPING

This Document is Confidential.

TABLE 5.16: DURATION OF iNO ADMINISTRATION BY TREATMENT ARM (5)

INTENTION-TO-TREAT POPULATION

		Treatment Arm		
		Arm A	Arm B	Total
Duration (days)	N			
	Missing			
	Mean			
	SD			
	Median			
	Minimum			
	First quartile			
	Third quartile			
	Maximum			

NOTE: Arm A = UMBELICAL CORD MILKING, Arm B = DELAYED UMBELICAL CORD CLAMPING

This Document is Confidential.

TABLE 5.17: DISTRIBUTION OF PATIENTS BY NEED OF ERYTHROCYTE CONCENTRATE TRANSFUSIONS AND TREATMENT ARM (1)

INTENTION-TO-TREAT POPULATION

	Treatment Arm				Total	
	Arm A		Arm B			
	N	%	N	%	N	%
Blood transfusions						
No						
Yes						
Missing value						
Total						

NOTE: Arm A = UMBELICAL CORD MILKING, Arm B = DELAYED UMBELICAL CORD CLAMPING

This Document is Confidential.

TABLE 5.18: NUMBER OF ERYTHROCYTE CONCENTRATE TRANSFUSION BY TREATMENT ARM (5)

INTENTION-TO-TREAT POPULATION

		Treatment Arm		
		Arm A	Arm B	Total
Number of blood transfusions	N			
	Missing			
	Mean			
	SD			
	Median			
	Minimum			
	First quartile			
	Third quartile			
	Maximum			

NOTE: Arm A = UMBELICAL CORD MILKING, Arm B = DELAYED UMBELICAL CORD CLAMPING

This Document is Confidential.

TABLE 5.19: DISTRIBUTION OF PATIENTS BY USE OF OXYGEN THERAPY AND TREATMENT ARM (1)

INTENTION-TO-TREAT POPULATION

	Treatment Arm				Total	
	Arm A		Arm B			
	N	%	N	%	N	%
Oxygen therapy						
No						
Yes						
Missing value						
Total						

NOTE: Arm A = UMBELICAL CORD MILKING, Arm B = DELAYED UMBELICAL CORD CLAMPING

This Document is Confidential.

TABLE 5.20: DURATION OF OXYGEN THERAPY BY TREATMENT ARM (5)

INTENTION-TO-TREAT POPULATION

		Treatment Arm		Total
		Arm A	Arm B	
Duration (days)	N			
	Missing			
	Mean			
	SD			
	Median			
	Minimum			
	First quartile			
	Third quartile			
	Maximum			

NOTE: Arm A = UMBELICAL CORD MILKING, Arm B = DELAYED UMBELICAL CORD CLAMPING

TABLE 5.21: FiO2 MAX DURING OXYGEN THERAPY BY TREATMENT ARM (5)

INTENTION-TO-TREAT POPULATION

		Treatment Arm		Total
		Arm A	Arm B	
FiO2 max (%)	N			
	Missing			
	Mean			
	SD			
	Median			
	Minimum			
	First quartile			
	Third quartile			
	Maximum			

NOTE: Arm A = UMBELICAL CORD MILKING, Arm B = DELAYED UMBELICAL CORD CLAMPING

This Document is Confidential.

TABLE 5.22: DISTRIBUTION OF PATIENTS BY DOPAMINE USE IN THE FIRST 24 HOURS AND TREATMENT ARM (1)

INTENTION-TO-TREAT POPULATION

	Treatment Arm				Total	
	Arm A		Arm B			
	N	%	N	%	N	%
Dopamine use						
No						
Yes						
Missing value						
Total						

NOTE: Arm A = UMBELICAL CORD MILKING, Arm B = DELAYED UMBELICAL CORD CLAMPING

This Document is Confidential.

TABLE 5.23: DISTRIBUTION OF PATIENTS BY DOBUTAMINE USE IN THE FIRST 24 HOURS AND TREATMENT ARM (1)

INTENTION-TO-TREAT POPULATION

	Treatment Arm				Total	
	Arm A		Arm B			
	N	%	N	%	N	%
Dobutamine use						
No						
Yes						
Missing value						
Total						

NOTE: Arm A = UMBELICAL CORD MILKING, Arm B = DELAYED UMBELICAL CORD CLAMPING

This Document is Confidential.

TABLE 5.24: OVERALL DURATION OF HOSPITALIZATION BY TREATMENT ARM (5)

INTENTION-TO-TREAT POPULATION

		Treatment Arm		Total
		Arm A	Arm B	
Duration (days)	N			
	Missing			
	Mean			
	SD			
	Median			
	Minimum			
	First quartile			
	Third quartile			
	Maximum			

NOTE: Arm A = UMBELICAL CORD MILKING, Arm B = DELAYED UMBELICAL CORD CLAMPING

This Document is Confidential.

TABLE 5.25: CEREBRAL OXYGENATION AT 3 HOURS BY TREATMENT ARM (5)

INTENTION-TO-TREAT POPULATION

		Treatment Arm		
		Arm A	Arm B	Total
3 hrs cerebral oxygenation	N			
	Missing			
	Mean			
	SD			
	Median			
	Minimum			
	First quartile			
	Third quartile			
	Maximum			

NOTE: Arm A = UMBELICAL CORD MILKING, Arm B = DELAYED UMBELICAL CORD CLAMPING

This Document is Confidential.

TABLE 5.26: CEREBRAL OXYGENATION AT 6 HOURS BY TREATMENT ARM (5)

INTENTION-TO-TREAT POPULATION

		Treatment Arm		
		Arm A	Arm B	Total
6 hrs cerebral oxygenation	N			
	Missing			
	Mean			
	SD			
	Median			
	Minimum			
	First quartile			
	Third quartile			
	Maximum			

NOTE: Arm A = UMBELICAL CORD MILKING, Arm B = DELAYED UMBELICAL CORD CLAMPING

This Document is Confidential.

TABLE 5.27: CEREBRAL OXYGENATION AT 12 HOURS BY TREATMENT ARM (5)

INTENTION-TO-TREAT POPULATION

		Treatment Arm		Total
		Arm A	Arm B	
12 hrs cerebral oxygenation	N			
	Missing			
	Mean			
	SD			
	Median			
	Minimum			
	First quartile			
	Third quartile			
	Maximum			

NOTE: Arm A = UMBELICAL CORD MILKING, Arm B = DELAYED UMBELICAL CORD CLAMPING

This Document is Confidential.

TABLE 5.28: CEREBRAL OXYGENATION AT 18 HOURS BY TREATMENT ARM (5)

INTENTION-TO-TREAT POPULATION

		Treatment Arm		Total
		Arm A	Arm B	
18 hrs cerebral oxygenation	N			
	Missing			
	Mean			
	SD			
	Median			
	Minimum			
	First quartile			
	Third quartile			
	Maximum			

NOTE: Arm A = UMBELICAL CORD MILKING, Arm B = DELAYED UMBELICAL CORD CLAMPING

This Document is Confidential.

TABLE 5.29: CEREBRAL OXYGENATION AT 24 HOURS BY TREATMENT ARM (5)

INTENTION-TO-TREAT POPULATION

		Treatment Arm		
		Arm A	Arm B	Total
24 hrs cerebral oxygenation	N			
	Missing			
	Mean			
	SD			
	Median			
	Minimum			
	First quartile			
	Third quartile			
	Maximum			

NOTE: Arm A = UMBELICAL CORD MILKING, Arm B = DELAYED UMBELICAL CORD CLAMPING

This Document is Confidential.

TABLE 5.30: SUPERIOR VENA CAVA BLOOD FLOW IN THE FIRST 24 HOURS BY TREATMENT ARM (5)

INTENTION-TO-TREAT POPULATION

		Treatment Arm		Total
		Arm A	Arm B	
Superior vena cava blood flow (ml/Kg/min)	N			
	Missing			
	Mean			
	SD			
	Median			
	Minimum			
	First quartile			
	Third quartile			
	Maximum			

NOTE: Arm A = UMBELICAL CORD MILKING, Arm B = DELAYED UMBELICAL CORD CLAMPING

This Document is Confidential.

TABLE 5.31: PAP VALUE BY TREATMENT ARM (5)

INTENTION-TO-TREAT POPULATION

		Treatment Arm		Total
		Arm A	Arm B	
PAP value (mmHg)	N			
	Missing			
	Mean			
	SD			
	Median			
	Minimum			
	First quartile			
	Third quartile			
	Maximum			

NOTE: Arm A = UMBELICAL CORD MILKING, Arm B = DELAYED UMBELICAL CORD CLAMPING

This Document is Confidential.

TABLE 5.32: RIGHT VENTRICULAR EJECTION BY TREATMENT ARM (5)

INTENTION-TO-TREAT POPULATION

		Treatment Arm		
		Arm A	Arm B	Total
Right ventricular ejection (ml/Kg/min)	N			
	Missing			
	Mean			
	SD			
	Median			
	Minimum			
	First quartile			
	Third quartile			
	Maximum			

NOTE: Arm A = UMBELICAL CORD MILKING, Arm B = DELAYED UMBELICAL CORD CLAMPING

This Document is Confidential.

TABLE 5.33: LEFT VENTRICULAR EJECTION BY TREATMENT ARM (5)

INTENTION-TO-TREAT POPULATION

		Treatment Arm		
		Arm A	Arm B	Total
Left ventricular ejection (ml/Kg/min)	N			
	Missing			
	Mean			
	SD			
	Median			
	Minimum			
	First quartile			
	Third quartile			
	Maximum			

NOTE: Arm A = UMBELICAL CORD MILKING, Arm B = DELAYED UMBELICAL CORD CLAMPING

This Document is Confidential.

TABLE 5.34: DISTRIBUTION OF PATIENTS BY TRANSDUCTAL SHUNT AND TREATMENT ARM (1)

INTENTION-TO-TREAT POPULATION

	Treatment Arm				Total	
	Arm A		Arm B			
	N	%	N	%	N	%
Transductal shunt						
Left-right						
Right-left						
Bidirectional						
Missing value						
Total						

NOTE: Arm A = UMBELICAL CORD MILKING, Arm B = DELAYED UMBELICAL CORD CLAMPING

This Document is Confidential.

TABLE 5.35: Hb MAX IN THE FIRST 24 HOURS BY TREATMENT ARM (5)

INTENTION-TO-TREAT POPULATION

		Treatment Arm		
		Arm A	Arm B	Total
Hb max (g/dl)	N			
	Missing			
	Mean			
	SD			
	Median			
	Minimum			
	First quartile			
	Third quartile			
	Maximum			

NOTE: Arm A = UMBELICAL CORD MILKING, Arm B = DELAYED UMBELICAL CORD CLAMPING

This Document is Confidential.

TABLE 5.36: Ht MAX IN THE FIRST 24 HOURS BY TREATMENT ARM (5)

INTENTION-TO-TREAT POPULATION

		Treatment Arm		Total
		Arm A	Arm B	
Ht max (%)	N			
	Missing			
	Mean			
	SD			
	Median			
	Minimum			
	First quartile			
	Third quartile			
	Maximum			

NOTE: Arm A = UMBELICAL CORD MILKING, Arm B = DELAYED UMBELICAL CORD CLAMPING

This Document is Confidential.

TABLE 5.37: AVERAGE PAS AT 3 HOURS BY TREATMENT ARM (5)

INTENTION-TO-TREAT POPULATION

		Treatment Arm		
		Arm A	Arm B	Total
3 hrs average PAS (mmHg)	N			
	Missing			
	Mean			
	SD			
	Median			
	Minimum			
	First quartile			
	Third quartile			
	Maximum			

NOTE: Arm A = UMBELICAL CORD MILKING, Arm B = DELAYED UMBELICAL CORD CLAMPING

This Document is Confidential.

TABLE 5.38: AVERAGE PAS AT 6 HOURS BY TREATMENT ARM (5)

INTENTION-TO-TREAT POPULATION

		Treatment Arm		Total
		Arm A	Arm B	
6 hrs average PAS (mmHg)	N			
	Missing			
	Mean			
	SD			
	Median			
	Minimum			
	First quartile			
	Third quartile			
	Maximum			

NOTE: Arm A = UMBELICAL CORD MILKING, Arm B = DELAYED UMBELICAL CORD CLAMPING

This Document is Confidential.

TABLE 5.39: AVERAGE PAS AT 12 HOURS BY TREATMENT ARM (5)

INTENTION-TO-TREAT POPULATION

		Treatment Arm		
		Arm A	Arm B	Total
12 hrs average PAS (mmHg)	N			
	Missing			
	Mean			
	SD			
	Median			
	Minimum			
	First quartile			
	Third quartile			
	Maximum			

NOTE: Arm A = UMBELICAL CORD MILKING, Arm B = DELAYED UMBELICAL CORD CLAMPING

This Document is Confidential.

TABLE 5.40: AVERAGE PAS AT 18 HOURS BY TREATMENT ARM (5)

INTENTION-TO-TREAT POPULATION

		Treatment Arm		
		Arm A	Arm B	Total
18 hrs average PAS (mmHg)	N			
	Missing			
	Mean			
	SD			
	Median			
	Minimum			
	First quartile			
	Third quartile			
	Maximum			

NOTE: Arm A = UMBELICAL CORD MILKING, Arm B = DELAYED UMBELICAL CORD CLAMPING

This Document is Confidential.

TABLE 5.41: AVERAGE PAS AT 24 HOURS BY TREATMENT ARM (5)

INTENTION-TO-TREAT POPULATION

		Treatment Arm		Total
		Arm A	Arm B	
24 hrs average PAS (mmHg)	N			
	Missing			
	Mean			
	SD			
	Median			
	Minimum			
	First quartile			
	Third quartile			
	Maximum			

NOTE: Arm A = UMBELICAL CORD MILKING, Arm B = DELAYED UMBELICAL CORD CLAMPING

This Document is Confidential.

TABLE 5.42: S/D AMS BY TREATMENT ARM (5)

INTENTION-TO-TREAT POPULATION

		Treatment Arm		Total
		Arm A	Arm B	
S/D AMS	N			
	Missing			
	Mean			
	SD			
	Median			
	Minimum			
	First quartile			
	Third quartile			
	Maximum			

NOTE: Arm A = UMBELICAL CORD MILKING, Arm B = DELAYED UMBELICAL CORD CLAMPING

This Document is Confidential.

TABLE 5.43: PI AMS BY TREATMENT ARM (5)

INTENTION-TO-TREAT POPULATION

		Treatment Arm		Total
		Arm A	Arm B	
PI AMS	N			
	Missing			
	Mean			
	SD			
	Median			
	Minimum			
	First quartile			
	Third quartile			
	Maximum			

NOTE: Arm A = UMBELICAL CORD MILKING, Arm B = DELAYED UMBELICAL CORD CLAMPING

This Document is Confidential.

TABLE 5.44: RI AMS BY TREATMENT ARM (5)

INTENTION-TO-TREAT POPULATION

		Treatment Arm		
		Arm A	Arm B	Total
RI AMS	N			
	Missing			
	Mean			
	SD			
	Median			
	Minimum			
	First quartile			
	Third quartile			
	Maximum			

NOTE: Arm A = UMBELICAL CORD MILKING, Arm B = DELAYED UMBELICAL CORD CLAMPING

This Document is Confidential.

TABLE 5.45: DISTRIBUTION OF PATIENTS BY AMS DIASTOLIC FLOW AND TREATMENT ARM (1)

INTENTION-TO-TREAT POPULATION

	Treatment Arm				Total	
	Arm A		Arm B			
	N	%	N	%	N	%
AMS diastolic flow						
Absent						
Present						
Unknown						
Missing value						
Total						

NOTE: Arm A = UMBELICAL CORD MILKING, Arm B = DELAYED UMBELICAL CORD CLAMPING

This Document is Confidential.

TABLE 5.46: S/D ACA BY TREATMENT ARM (5)

INTENTION-TO-TREAT POPULATION

		Treatment Arm		Total
		Arm A	Arm B	
S/D ACA	N			
	Missing			
	Mean			
	SD			
	Median			
	Minimum			
	First quartile			
	Third quartile			
	Maximum			

NOTE: Arm A = UMBELICAL CORD MILKING, Arm B = DELAYED UMBELICAL CORD CLAMPING

This Document is Confidential.

TABLE 5.47: PI ACA BY TREATMENT ARM (5)

INTENTION-TO-TREAT POPULATION

		Treatment Arm		Total
		Arm A	Arm B	
PI ACA	N			
	Missing			
	Mean			
	SD			
	Median			
	Minimum			
	First quartile			
	Third quartile			
	Maximum			

NOTE: Arm A = UMBELICAL CORD MILKING, Arm B = DELAYED UMBELICAL CORD CLAMPING

This Document is Confidential.

TABLE 5.48: RI ACA BY TREATMENT ARM (5)

INTENTION-TO-TREAT POPULATION

		Treatment Arm		Total
		Arm A	Arm B	
RI ACA	N			
	Missing			
	Mean			
	SD			
	Median			
	Minimum			
	First quartile			
	Third quartile			
	Maximum			

NOTE: Arm A = UMBELICAL CORD MILKING, Arm B = DELAYED UMBELICAL CORD CLAMPING

This Document is Confidential.

TABLE 5.49: DISTRIBUTION OF PATIENTS BY ACA DIASTOLIC FLOW AND TREATMENT ARM (1)

INTENTION-TO-TREAT POPULATION

	Treatment Arm				Total	
	Arm A		Arm B			
	N	%	N	%	N	%
ACA diastolic flow						
Absent						
Present						
Unknown						
Missing value						
Total						

NOTE: Arm A = UMBELICAL CORD MILKING, Arm B = DELAYED UMBELICAL CORD CLAMPING

This Document is Confidential.

6. SAFETY ANALYSES

TABLE 6.1: DISTRIBUTION OF PATIENTS BY INTUBATION IN DELIVERY ROOM AND TREATMENT ARM (1)

SAFETY POPULATION

	Treatment Arm				Total	
	Arm A		Arm B			
	N	%	N	%	N	%
Intubation						
No						
Yes						
Missing value						
Total						

NOTE: Arm A = UMBELICAL CORD MILKING, Arm B = DELAYED UMBELICAL CORD CLAMPING

This Document is Confidential.

TABLE 6.2: DISTRIBUTION OF PATIENTS BY COMPRESSIONS IN DELIVERY ROOM AND TREATMENT ARM (1)

SAFETY POPULATION

	Treatment Arm				Total	
	Arm A		Arm B			
	N	%	N	%	N	%
Compressions						
No						
Yes						
Missing value						
Total						

NOTE: Arm A = UMBELICAL CORD MILKING, Arm B = DELAYED UMBELICAL CORD CLAMPING

This Document is Confidential.

TABLE 6.3: DISTRIBUTION OF PATIENTS BY USE OF IV ADRENALINE IN DELIVERY ROOM AND TREATMENT ARM (1)

SAFETY POPULATION

	Treatment Arm				Total	
	Arm A		Arm B			
	N	%	N	%	N	%
IV adrenaline						
No						
Yes						
Missing value						
Total						

NOTE: Arm A = UMBELICAL CORD MILKING, Arm B = DELAYED UMBELICAL CORD CLAMPING

This Document is Confidential.

TABLE 6.4: DISTRIBUTION OF PATIENTS BY USE OF ET ADRENALINE IN DELIVERY ROOM AND TREATMENT ARM (1)

SAFETY POPULATION

	Treatment Arm				Total	
	Arm A		Arm B			
	N	%	N	%	N	%
ET adrenaline						
No						
Yes						
Missing value						
Total						

NOTE: Arm A = UMBELICAL CORD MILKING, Arm B = DELAYED UMBELICAL CORD CLAMPING

This Document is Confidential.

TABLE 6.5: DISTRIBUTION OF PATIENTS BY USE OF SURFACTANT IN DELIVERY ROOM AND TREATMENT ARM (1)

SAFETY POPULATION

	Treatment Arm				Total	
	Arm A		Arm B			
	N	%	N	%	N	%
Surfactant						
No						
Yes						
Missing value						
Total						

NOTE: Arm A = UMBELICAL CORD MILKING, Arm B = DELAYED UMBELICAL CORD CLAMPING

This Document is Confidential.

TABLE 6.6: TOTAL NUMBER OF SURFACTANT DOSES BY TREATMENT ARM (5)

SAFETY POPULATION

		Treatment Arm		
		Arm A	Arm B	Total
Surfactant doses	N			
	Missing			
	Mean			
	SD			
	Median			
	Minimum			
	First quartile			
	Third quartile			
	Maximum			

NOTE: Arm A = UMBELICAL CORD MILKING, Arm B = DELAYED UMBELICAL CORD CLAMPING

This Document is Confidential.

TABLE 6.7: CRIB II SCORE BY TREATMENT ARM (5)

SAFETY POPULATION

		Treatment Arm		Total
		Arm A	Arm B	
CRIB II score	N			
	Missing			
	Mean			
	SD			
	Median			
	Minimum			
	First quartile			
	Third quartile			
	Maximum			

NOTE: Arm A = UMBELICAL CORD MILKING, Arm B = DELAYED UMBELICAL CORD CLAMPING

This Document is Confidential.

TABLE 6.8: MAX SERUM BILIRUBIN BY TREATMENT ARM (5)

SAFETY POPULATION

		Treatment Arm		Total
		Arm A	Arm B	
Max serum bilirubin	N			
	Missing			
	Mean			
	SD			
	Median			
	Minimum			
	First quartile			
	Third quartile			
	Maximum			

NOTE: Arm A = UMBELICAL CORD MILKING, Arm B = DELAYED UMBELICAL CORD CLAMPING

This Document is Confidential.

TABLE 6.9: DISTRIBUTION OF PATIENTS BY EXCHANGE TRANSFUSION AND TREATMENT ARM (1)

SAFETY POPULATION

	Treatment Arm				Total	
	Arm A		Arm B			
	N	%	N	%	N	%
Exchange transfusions						
No						
Yes						
Missing value						
Total						

NOTE: Arm A = UMBELICAL CORD MILKING, Arm B = DELAYED UMBELICAL CORD CLAMPING

This Document is Confidential.

TABLE 6.10: DISTRIBUTION OF PATIENTS BY NEED OF MECHANICAL VENTILATION IN THE FIRST 24 HOURS AND TREATMENT ARM (1)

SAFETY POPULATION

	Treatment Arm				Total	
	Arm A		Arm B			
	N	%	N	%	N	%
Mechanical ventilation						
No						
Yes						
Missing value						
Total						

NOTE: Arm A = UMBELICAL CORD MILKING, Arm B = DELAYED UMBELICAL CORD CLAMPING

This Document is Confidential.

TABLE 6.11: DURATION OF MECHANICAL VENTILATION IN THE FIRST 24 HOURS BY TREATMENT ARM (5)

SAFETY POPULATION

		Treatment Arm		
		Arm A	Arm B	Total
Duration of MV (hrs)	N			
	Missing			
	Mean			
	SD			
	Median			
	Minimum			
	First quartile			
	Third quartile			
	Maximum			

NOTE: Arm A = UMBELICAL CORD MILKING, Arm B = DELAYED UMBELICAL CORD CLAMPING

This Document is Confidential.

TABLE 6.12: DISTRIBUTION OF PATIENTS BY PHARMACOLOGICAL CLOSURE OF PDA AND TREATMENT ARM (1)

SAFETY POPULATION

	Treatment Arm				Total	
	Arm A		Arm B			
	N	%	N	%	N	%
Pharmacological closure of PDA						
No						
Yes						
Missing value						
Total						

NOTE: Arm A = UMBELICAL CORD MILKING, Arm B = DELAYED UMBELICAL CORD CLAMPING

This Document is Confidential.

TABLE 6.13: DISTRIBUTION OF PATIENTS BY NECROTIZING ENTEROCOLITIS AND TREATMENT ARM (1)

SAFETY POPULATION

	Treatment Arm				Total	
	Arm A		Arm B			
	N	%	N	%	N	%
NEC						
No						
Yes						
Missing value						
Total						

NOTE: Arm A = UMBELICAL CORD MILKING, Arm B = DELAYED UMBELICAL CORD CLAMPING

This Document is Confidential.

TABLE 6.14: DISTRIBUTION OF PATIENTS BY PERIVENTRICULAR LEUKOMALACIA AND TREATMENT ARM (1)

SAFETY POPULATION

	Treatment Arm				Total	
	Arm A		Arm B			
	N	%	N	%	N	%
PLV						
No						
Yes						
Missing value						
Total						

NOTE: Arm A = UMBELICAL CORD MILKING, Arm B = DELAYED UMBELICAL CORD CLAMPING

This Document is Confidential.

TABLE 6.15: DISTRIBUTION OF PATIENTS BY INTRAVENTRICULAR HEMORRHAGE AND TREATMENT ARM (1)

SAFETY POPULATION

	Treatment Arm				Total	
	Arm A		Arm B			
	N	%	N	%	N	%
IVH						
No IVH						
Grade 1						
Grade 2						
Grade 3						
Grade 4						
Missing value						
Total						

NOTE: Arm A = UMBELICAL CORD MILKING, Arm B = DELAYED UMBELICAL CORD CLAMPING

This Document is Confidential.

TABLE 6.16: DISTRIBUTION OF PATIENTS BY RETINOPATHY OF PREMATURITY AND TREATMENT ARM (1)

SAFETY POPULATION

	Treatment Arm				Total	
	Arm A		Arm B			
	N	%	N	%	N	%
ROP						
No						
Yes						
Missing value						
Total						

NOTE: Arm A = UMBELICAL CORD MILKING, Arm B = DELAYED UMBELICAL CORD CLAMPING

This Document is Confidential.

TABLE 6.17: DISTRIBUTION OF PATIENTS BY SEPSIS AND TREATMENT ARM (1)

SAFETY POPULATION

	Treatment Arm				Total	
	Arm A		Arm B			
	N	%	N	%	N	%
Sepsis						
No						
Yes, early onset						
Yes, late onset						
Missing value						
Total						

NOTE: Arm A = UMBELICAL CORD MILKING, Arm B = DELAYED UMBELICAL CORD CLAMPING

This Document is Confidential.
