# Supplementary material for: Resuscitation With Placental Circulation Intact Compared With Cord Milking: A Randomized Clinical Trial
Source: JAMA Netw Open. 2024 Dec 13;7(12):e2450476. doi: 10.1001/jamanetworkopen.2024.50476 (PMC11645650; doi:10.1001/jamanetworkopen.2024.50476)
Supplement: Supplement 2. — eTable 1. Primary composite outcome overall and in prespecified subgroups (per protocol), rate and (%) eTable 2. Per protocol analysis of grade 3-4 IVH, death, and bronchopulmonary dysplasia, rate and (%) eTable 3. Secondary outcomes by treatment group (per protocol) [file jamanetwopen-e2450476-s002.pdf]

Supplemental Online Content

Pratesi S, Boni L, Ciarcià M, et al; PCI Trial Collaborators. Resuscitation with placental circulation intact compared with cord milking: a randomized clinical trial. *JAMA Netw Open*. 2024;7(12):e2450476. doi:10.1001/jamanetworkopen.2024.50476

eTable 1. Primary composite outcome overall and in prespecified subgroups (per protocol), rate and (%)

eTable 2. Per protocol analysis of grade 3-4 IVH, death, and bronchopulmonary dysplasia rate and (%)

eTable 3. Secondary outcomes by treatment group (per protocol)

This supplemental material has been provided by the authors to give readers additional information about their work.

© 2024 Pratesi S et al. *JAMA Network Open.*

eTable 1. Primary composite outcome overall and in prespecified subgroups (per protocol), rate and (%)

	**UCM (n=99)**	**PCI (n=85)**	**OR (95% C.I.)**
Overall	36/99 (36)	23/85 (27)	0.65 (0.34-1.21)
23^{+0}-26^{+6} wks	25/43 (58)	14/30 (47)	0.63 (0.24-1.61)
Death	9/43 (21)	2/30 (7)	0.60 (0.20-1.73)
grade 3-4 IVH	10/43 (23)	3/30 (10)	0.60 (0.20-1.73)
BPD	15/43 (35)	11/30 (37)	1.08 (0.40-2.85)
27^{+0}-29^{+6} wks	11/56 (20)	9/55 (16)	0.80 (0.30-2.11)
Death	2/56 (4)	2/55 (4)	0.95 (0.12-6.97)
grade 3-4 IVH	1/56 (2)	1/55 (2)	0.95 (0.05-15.55)
BPD	8/56 (14)	6/55 (11)	0.73 (0.23-2.27)
Vaginal delivery	14/38 (37)	11/39 (28)	0.62 (0.27-1.45)
Death	5/38 (13)	2/39 (5)	0.59 (0.15-2.38)
grade 3-4 IVH	6/38 (16)	4/39 (10)	0.90 (0.27-2.93)
BPD	8/38 (21)	7/39 (18)	0.82 (0.34-2.11)
Cesarean section	22/61 (36)	12/46 (26)	0.67 (0.25-1.75)
Death	6/61 (10)	2/46 (4)	0.71 (0.21-2.32)
grade 3-4 IVH	5/61 (8)	0/46 (0)	0.18 (0.02-1.54)
BPD	15/61 (25)	10/46 (22)	0.85 (0.34-2.11)

Abbreviations: UCM, umbilical cord milking; PCI, placental circulation intact; OR, odds ratio; BPD, bronchopulmonary dysplasia; IVH, intraventricular hemorrhage

© 2024 Pratesi S et al. *JAMA Network Open.*

eTable 2. Per protocol analysis of grade 3-4 IVH, death, and bronchopulmonary dysplasia, rate and (%)

	UCM (n=99)	PCI (n=85)	OR (95% CI)
grade 3-4 IVH	11/99 (11)	4/85 (5)	0.39 (0.12-1.29)
Death	11/99 (11)	4/85 (5)	0.39 (0.12-1.29)
BPD	23/99 (23)	17/85 (20)	0.82 (0.40-1.67)

Abbreviations: UCM, umbilical cord milking; PCI, placental circulation intact; OR, odds ratio; BPD, bronchopulmonary dysplasia; IVH, intraventricular hemorrhage

© 2024 Pratesi S et al. *JAMA Network Open.*

eTable 3. Secondary outcomes by treatment group (per protocol)

	**UCM (n=99)**	**PCI (n=85)**	**P**
Hb max first 24h of life (g/dl), median (IQR: Q1, Q3)	17.7 (16-20.5)	18.4 (16-20.7)	0.39
Ht max first 24h of life (%), median (IQR: Q1, Q3)	52 (46-59)	53 (47-61)	0.42
Need of Blood Transfusion, No (%)	73 (74)	58 (68)	0.41
Number of Blood Transfusion, median (IQR: Q1, Q3)	2 (0-4)	1 (0-3)	0.29
Mechanical ventilation in first 24h of life, No (%)	35 (34)	39 (46)	0.14
Non invasive ventilation, No (%)	90 (91)	81 (95)	0.24
Mechanical ventilation, No (%)	46 (46)	48 (56)	0.17
INO therapy, No (%)	16 (16)	14 (16)	0.95
PDA treatment, No. (%)	49 (49)	42 (49)	0.99
Lenght of stay, median (IQR: Q1, Q3), days	81 (54-100)	78 (56-104)	0.78
Peak serum BR, median (IQR: Q1, Q3), mg/dL	8.5 (7-10.4)	8.5 (6.6-10.2)	0.34
NEC, No (%)	2 (2.0)	2 (2.3)	0.87
PVL, No (%)	4 (4.0)	2 (2.3)	0.52
IVH 1+2, No (%)	12 (12)	15 (18)	0.33
ROP, No (%)	27 (27)	19 (22)	0.44
Early onset Sepsis, No (%)	8 (8)	2 (2.3)	0.16
Late onset Sepsis, No (%)	34 (34)	26 (31)	0.24

Abbreviations: UCM, umbilical cord milking; PCI, placental circulation intact; OR, odds ratio; BPD, bronchopulmonary dysplasia; IVH, intraventricular hemorrhage; PVL, periventricular leukomalacia; NEC, necrotizing enterocolitis

© 2024 Pratesi S et al. *JAMA Network Open.*
