# Supplementary material for: Resuscitation With Placental Circulation Intact Compared With Cord Milking: A Randomized Clinical Trial
Source: JAMA Netw Open. 2024 Dec 13;7(12):e2450476. doi: 10.1001/jamanetworkopen.2024.50476 (PMC11645650; doi:10.1001/jamanetworkopen.2024.50476)
Supplement: Supplement 3. — PCI Trial Collaborators [file jamanetwopen-e2450476-s003.pdf]

Supplemental Online Content: Nonauthor Collaborators
*First name, last name, and suffix (if applicable) are required and will appear in PubMed.

*Group Name(s): PCI Trial Collaborators							
***First Name and Middle Initial(s)**	***Last Name**	***Suffix (eg, Jr, III)**	Academic Degrees	Institution	Location (city, state/province, country)	Role or Contribution, eg, chair, principal investigator	Group (if more than 1 Group listed in the byline) and/or Subgroup (eg, Steering Committee)
Silvia	Perugi		MD	Careggi University Hospital	Florence/Italy	clinical research coordinator	
Valentina	Leonardi		MD	Careggi University Hospital	Florence/Italy	clinical research coordinator	
Caterina	Coviello		MD	Careggi University Hospital	Florence/Italy	clinical research coordinator	
Daniele	Roncati		MD	Careggi University Hospital	Florence/Italy	clinical research coordinator	
Serena	Elia		MD	Careggi University Hospital	Florence/Italy	clinical research coordinator	
Alessandra	Cecchi		MD	Careggi University Hospital	Florence/Italy	clinical research coordinator	
Marta	Capocasale		MD	Di Venere" Hospital	Bari/Italy	clinical research coordinator	
Caterina	Franco		MD	Di Venere" Hospital	Bari/Italy	clinical research coordinator	
Genny	Gottardi		MD	San Bortolo Hospital	Vicenza, Italy	clinical research coordinator	
Alessandra	Grison		MD	San Bortolo Hospital	Vicenza, Italy	clinical research coordinator	
Valentina	Dal Cengio		MD	San Bortolo Hospital	Vicenza, Italy	clinical research coordinator	
Valentina	Vanzo		MD	San Bortolo Hospital	Vicenza, Italy	clinical research coordinator	
