# Supplementary material for: Resuscitation With Placental Circulation Intact Compared With Cord Milking: A Randomized Clinical Trial
Source: JAMA Netw Open. 2024 Dec 13;7(12):e2450476. doi: 10.1001/jamanetworkopen.2024.50476 (PMC11645650; doi:10.1001/jamanetworkopen.2024.50476)
Supplement: Supplement 4. — Data Sharing Statement [file jamanetwopen-e2450476-s004.pdf]

Data Sharing Statement

Pratesi. Resuscitation With Placental Circulation Intact Compared With Cord Milking. *JAMA Netw Open*. Published December 13, 2024. doi:10.1001/jamanetworkopen.2024.50476

Data
Additional Information: Clinicaltrials.gov identifier NCT02671305 (date of registration: 26 JAN 2016)
Data available: Yes
Data types: Deidentified participant data
How to access data: Luca.Boni@hsanmartino.it and simone.pratesi@unifi.it
When available: With publication

Supporting Documents
Document types: None

Additional Information
Who can access the data: researchers whose proposed use of the data has been approved
Types of analyses: for any purpose
Mechanisms of data availability: with investigator support and after approval of a proposal
